# Mesothelin as Biomarker and Therapeutic Target for Immunotherapy in Cancer

**DOI:** 10.1002/mco2.71009

**Published:** 2026-09-25

**Authors:** Manoj Kumar Gupta, Ramakrishna Vadde, Ganji Purnachandra Nagaraju

**Affiliations:** ^1^ Hematology, Hemostasis, Oncology, and Stem Cell Transplantation Hannover Medical School Hannover Germany; ^2^ Department of Biotechnology & Bioinformatics Yogi Vemana University Kadapa Andhra Pradesh India; ^3^ Division of Hematology & Oncology Heersink School of Medicine The University of Alabama at Birmingham Birmingham Alabama USA

**Keywords:** cancer, CAR‐T, immunotherapy, mesothelin, monoclonal antibodies

## Abstract

Cancer remains a critical global health concern due to late detection, drug resistance, and high mortality. Mesothelin (MSLN) is a glycoprotein highly expressed on the surface of various cancer cells, including those of ovarian, pancreatic, and gastric cancers. Thus, it could be a valuable target for both as a biomarker and for immunotherapy. This review summarizes MSLN biology in cancer, current immunotherapeutic strategies targeting MSLN, associated clinical challenges, and emerging avenues to enhance immunotherapy efficacy. During cancer progression, MSLN acts as a cellular accelerator, promoting cell division and survival. It activates several critical signaling pathways, including nuclear factor k‐light‐chain‐enhancer of activated B cells (NF‐κB), interleukin‐6 and its soluble receptor (IL‐6/sIL‐6R), and c‐Jun N‐terminal kinase (JNK), thereby promoting epithelial–mesenchymal transition and angiogenesis. Various MSLN‐targeting immunotherapies, including monoclonal antibodies, chimeric antigen receptor‐T cells, cytokine therapies, and vaccines, have shown preclinical and early clinical efficacy. However, immune evasion, an immunosuppressive tumor microenvironment, antigen heterogeneity, tumor immune escape, and off‐tumor toxicities continue to hinder the broad clinical success of these modalities. Nonetheless, rational combinations of MSLN‐directed therapies with other immunomodulatory strategies, optimized patient stratification, and advances in vaccine and adoptive cell therapy design hold promise for overcoming resistance and improving patient outcomes.

## Graphical abstract

Mesothelin overexpression activate important pathways like nuclear factor k‐light‐chain‐enhancer of activated B cells (NF‐κB) and interleukin‐6 (IL‐6). This in turn causes numerous cancers, such as pancreatic and ovarian cancers. MSLN‐targeting immunotherapies, like monoclonal antibodies, have shown efficacy. However, immune evasion, an immunosuppressive tumor microenvironment, and antigen heterogeneity hinder the broad clinical success of these modalities. Nonetheless, combinations of MSLN‐directed therapies with other immunomodulatory strategies have shown preclinical efficacy.

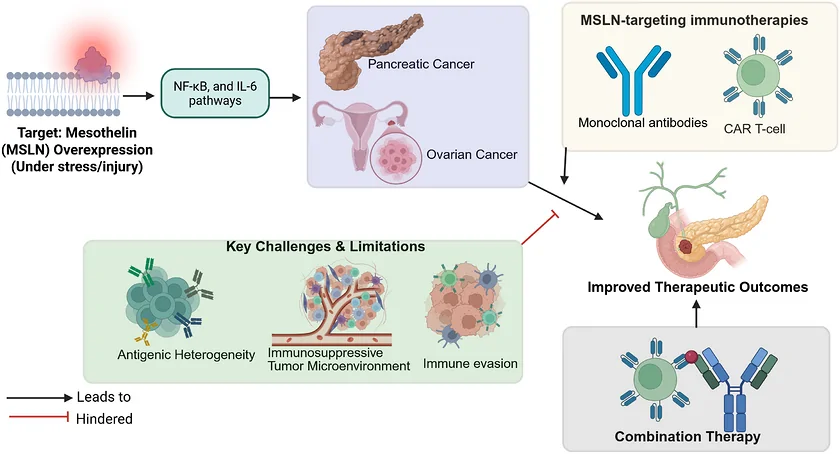

## 1 Introduction

**FIGURE 1:**
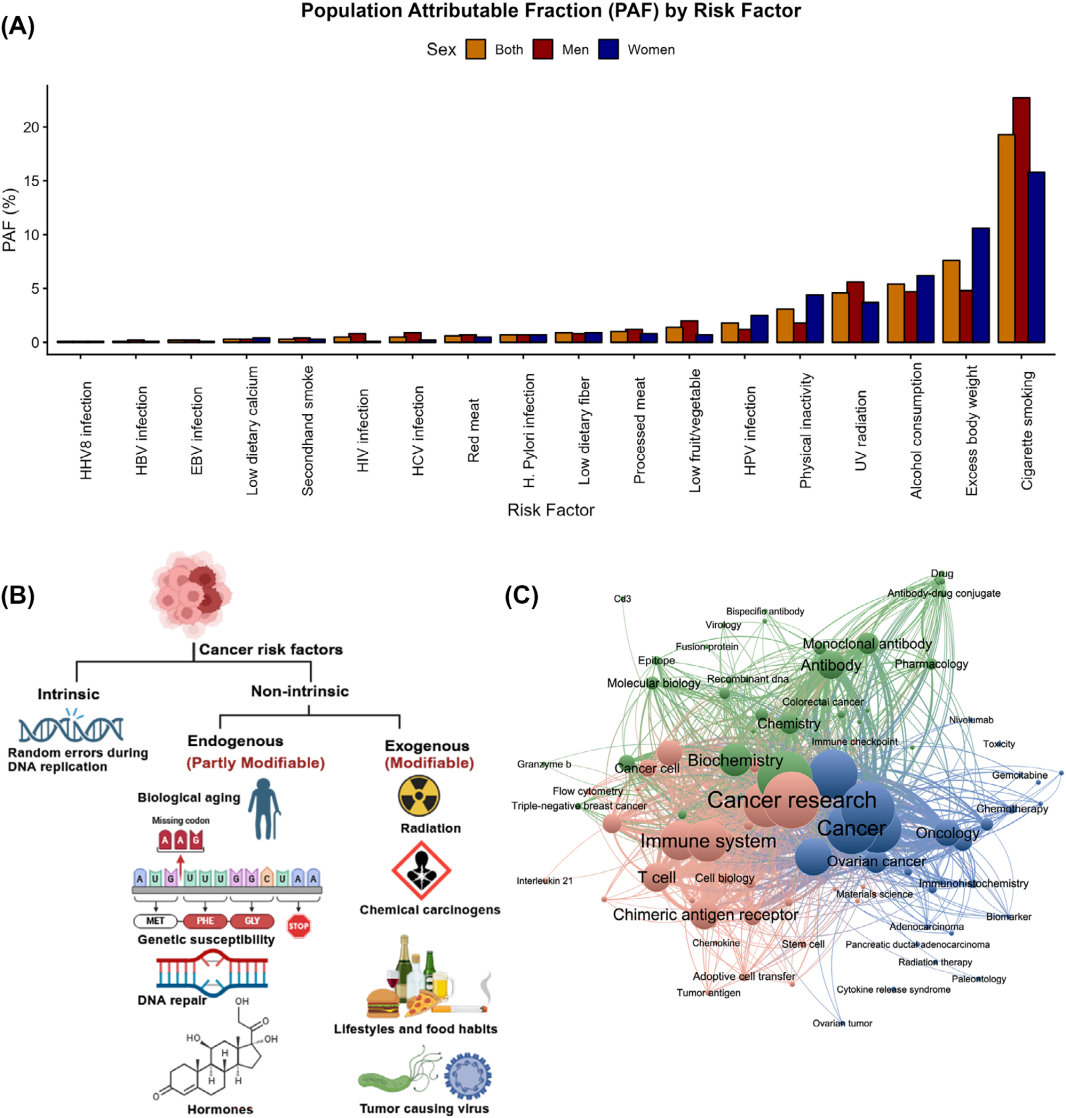
Estimated cancer burden, risk factors, and MSLN‐related research landscape. (A) Estimated proportion and number of cancer cases attributable to evaluated risk factors among U.S. adults aged 30 years and older in 2014, by sex. Population attributable fractions (PAFs) shown on the x‐axis represent the percentage of incident cancer cases attributable to the evaluated risk factors. The total number of incident cancer cases (excluding nonmelanoma skin cancer) was 782,210 in men, 788,765 in women, and 1,570,975 overall [3]. (B) Cancer risk factors can be grouped into three types. Overall, they are divided into two mutually exclusive categories: intrinsic (unmodifiable) and non‐intrinsic (at least partially modifiable) risk factors. Intrinsic risk factors arise from random errors during DNA replication. Non‐intrinsic risk factors are further classified as endogenous or exogenous, depending on whether they originate from internal biological processes or external environmental exposures, respectively [3, 4]. (C) Co‐occurrence network of keywords from PubMed literature on *MSLN* and human diseases. This network shows thematic links identified through text mining of PubMed abstracts up to March 2026. The main node, “Cancer research,” (dark red) is the primary topic and iss directly linked to two major subdomains: “Cancer,” (blue) which covers tumor types and disease mechanisms, and “Biochemistry,” (green) which focuses on processes at the molecular and cellular levels. The size of a node shows how important a keyword is (frequency/centrality), and the thickness of an edge shows how strong the relationship is between two terms. The way the terms are grouped shows connections across fields, such as *MSLN* and immunotherapy (e.g., chimeric antigen receptor, monoclonal antibodies), oncology (e.g., ovarian cancer, chemotherapy), and molecular biology (e.g., recombinant DNA, fusion proteins). This shows how *MSLN* is a complex target in cancer biology and treatment. Biorender was used to generate (B). Values depicted in (A) have been taken from Ref. [3], and values depicted in (B) have been taken from Refs. [3] and [4], and were used to generate an original figure using the ggplot2 package in R (version 4.3.2). Ref. [3] has a CC BY‐NC‐ND 4.0 license, and Ref. [4] has a CC BY 4.0 license.

Cancer is a significant global public health problem [1]. In the United States, cancer is the second most common cause of death for people of all ages. However, among people aged 65–84 years old, cancer is the leading cause of death. Smoking, drinking, and infections are the leading causes of cancer [2, 3] (Figure 1A). Other factors include genetic mutations, impaired DNA repair, ultraviolet (UV) radiation, chemical exposures, high consumption of processed meat, and low consumption of fresh fruits and vegetables. These cancer‐associated risk factors can be broadly classified into two categories: intrinsic and nonintrinsic (Figure 1B). Intrinsic risk factors cannot be modified and may arise from random errors in DNA replication. Nonintrinsic risk factors, on the other hand, are at least partially modifiable and can be endogenous or exogenous, depending on whether they arise from internal or external causes. Nevertheless, the influence of these risk factors may also vary by sex (Figure 1A). For instance, a 2019 study observed that the cancer risk associated with smoking and second‐hand smoke was comparable between men and women, albeit marginally higher in men. Likewise, men were more likely than women to develop cancer from drinking alcohol, except for esophageal cancer. Diet‐related factors accounted for 4.9% of cancer cases in men and 3.4% in women. Likewise, 92.2% of melanoma was caused by UV exposure, with a slightly higher percentage in men (94.5%) than in women (88.8%). Women had a higher risk of anal cancer from human papillomaviruses, while men had a higher risk of cancer from other infections [3].

Cancer is a highly heterogeneous and complex disease that affects multiple pathways and biological processes, including persistent signaling for cell growth, evasion of programmed cell death, and the ability to invade adjacent tissues and disseminate to distant sites [5, 6]. However, these processes are regulated by two major classes of genes: tumor suppressor genes and oncogenes. The tumor suppressor genes, such as tumor protein p53 *(TP53)*  and retinoblastoma 1 (*RB1)*, are associated with cell cycle regulation, DNA repair, and apoptosis. They act as cellular “brakes” and limit aberrant cell division. On the other hand, oncogenes, such as mesothelin (*MSLN*), act as cellular accelerators. They promote cell division and survival [7, 8]. *MSLN* expression is associated with various types of solid tumors (Figure 1C), including mesotheliomas (MESOs), pancreatic, biliary tract, ovarian, lung, glioblastomas, thymic, and gastric cancers [9, 10, 11, 12, 13, 14].

Due to its higher expression across various cancers, *MSLN* is considered a promising primary therapeutic target [15], particularly for immunotherapy [8, 16]. The concept of harnessing the body's immune system to fight cancer emerged in the nineteenth century [17]. Among the earliest people who observed a relationship between the immune response and cancer were Wilhelm Busch and Friedrich Fehleisen. They observed spontaneous tumor regression in patients with erysipelas, a skin condition usually caused by *Streptococcus pyogenes* [18]. Subsequently, William Coley, considered the “Father of Cancer Immunotherapy,” discovered a link between erysipelas and improved outcomes in patients with sarcomas [19]. To establish this empirical observation, Coley injected cancer patients with heat‐killed *Streptococcus pyogenes* and *Serratia marcescens*, both of which were known to affect the immune system [20]. “Coley's toxins” were found to have considerable immunostimulatory properties, leading to positive outcomes in several cancer types [19]. However, the lack of proper methodology and reproducibility standards, along with the development of radiation treatments and chemotherapy, hindered the widespread implementation of the approach in cancer treatment [18].

The idea of immunotherapy for cancer treatment resurfaced in the twentieth century and was significantly advanced by technological advances. In 1909, Paul Ehrlich suggested that neoplastic cells were constantly produced within the human body but are usually destroyed by the immune system [17, 20]. Later, Lewis Thomas and Sir Frank Macfarlane Burnet independently developed the cancer immunosurveillance theory, suggesting that tumors produce neoantigens that are detected and destroyed by the immune system to prevent cancer development, a process similar to graft rejection [18]. The immune responses observed following the adoptive transfer of tumors into mice, along with the regression of spontaneous melanomas in patients with autoimmune diseases, further supported the immunosurveillance theory. However, the unifying principle that connected the two was unknown at the time. Knockout mice provided the means to prove that immunosuppression can lead to carcinogenesis [17]. Later, advances in molecular biology and biochemistry enabled the study of immunity to tumor cells. The results of such studies provided convincing proof that the immune system can detect tumor cells. Thus, cancer immunotherapy has become a breakthrough in the treatment of cancer; it allows people diagnosed with incurable cancer to live significantly longer.

The use of anti‐MSLN antibodies, antibody–drug conjugates (ADCs), and chimeric antigen receptor T‐cell therapy (CAR‐T) targeting MSLN has shown effective antitumor responses in preclinical and early‐stage clinical investigations [16]. In this context, early studies have also found that therapies targeting MSLN have low toxicity to normal cells. Therefore, there has been ongoing clinical investigation on the development of an antibody‐based system and vaccine for MSLN [13, 15]. However, these strategies still face many challenges, such as tumor‐specific toxicity and a suppressive tumor microenvironment (TME) [13]. Overall, these limitations make current therapies less clinically effective, underscoring the need for a deeper understanding of MSLN's role in cancer development and its ability to modulate the TME. Considering these issues, this review provides extensive insight into the biological properties of *MSLN* and its implications for therapeutic targeting. This includes gene structure and functions that promote tumor progression and metastasis. In addition, different treatments targeting MSLN and methods to improve their translational capacity have also been reviewed.

## 2 Biology of MSLN in Cancer

To fully leverage MSLN as a therapeutic target and diagnostic biomarker, a comprehensive understanding of its molecular biology, structural processing, and functional mechanisms is essential. The biological activity of MSLN is tightly regulated by its genetic architecture, posttranslational modifications, and membrane localization on tumor cells.

### 2.1 Gene and Protein Structure

**FIGURE 2:**
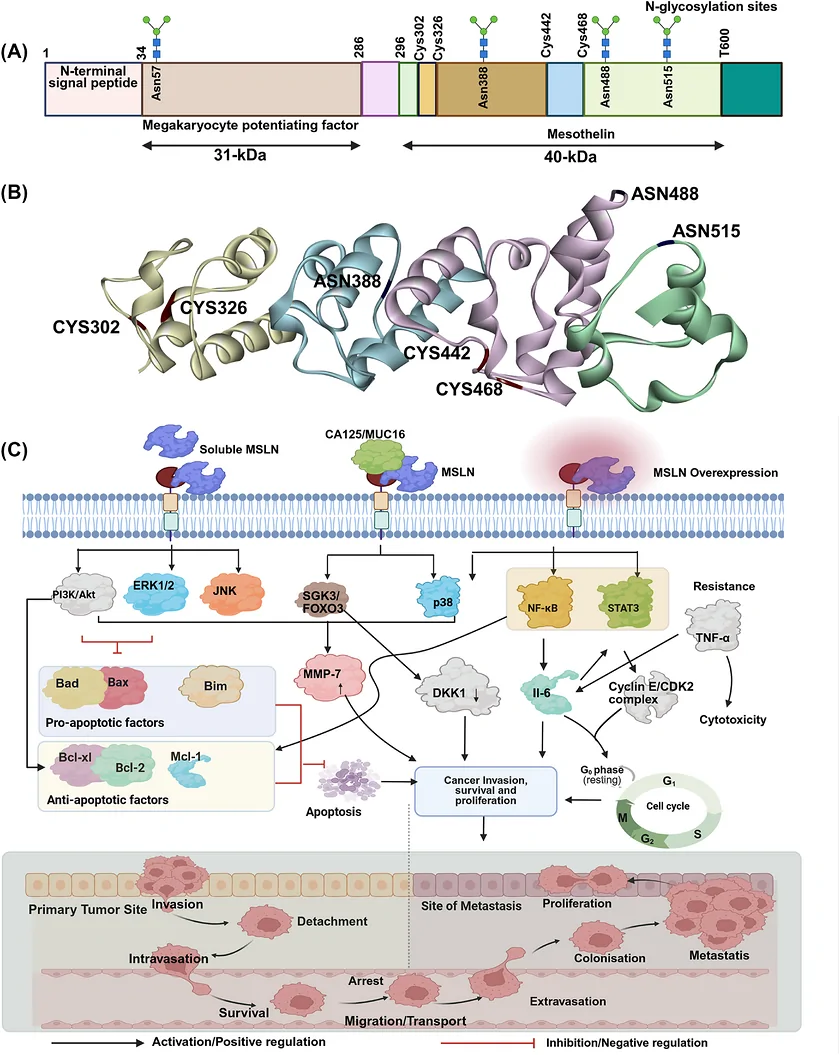
Structure and Oncogenic Signaling Pathways of Human MSLN. (A) The human MSLN gene is located on chromosome 16p13.3 [21, 25]. The figure represents the precursor protein of the major form (Q13421‐3) expressed by the human MSLN gene. This precursor protein, weighing 71 kDa, is proteolytically processed into two distinct proteins: one is the megakaryocyte potentiating factor, a 30 kDa protein containing residues Ser34‐Arg286, and the other is the mature, ∼41 kDa membrane‐bound mesothelin (starting at Glu296), which undergoes C‐terminal processing during GPI‐anchor attachment with removal of the C‐terminal signal peptide region (∼residues 599–622). Four N‐glycosylation sites have been identified in mesothelin at Asn57, Asn388, Asn488, and Asn515. Disulfide bridges conserved within the protein are Cys302‐Cys326 and Cys442‐Cys468. Furin cleavage occurs at Arg295 (B). The three‐dimensional structure of MSLN. This cartoon shows the crystal structure of MSLN (PDB ID: 8XC3). This protein comprises clear “natural” boundaries defined by four subdomains: A (aa296–359; golden), B (aa360–426; blue), C (aa427–501; grey), and D (aa502–585; pink). Asn388, Asn488, and Asn515 are present in domains B, C, and D, respectively. Cys302‐Cys326 is present in domain A, and Cys442‐Cys468 is present in domain C. The natural boundaries align well with recombinant fragments that are soluble or can be successfully refolded into a soluble state. The four‐domain architecture provides a larger binding surface during protein‐protein interactions. Domains A and B create the high‐affinity binding site for CA125. On the other hand, domains C and D, which are more proximal to the cell membrane, play roles in protein stability, glycosylation, association with GPI anchors, and protease recognition [13, 26]. (C) Mesothelin, whether in its soluble form or on the cell surface, activates several key signaling pathways, including Akt, ERK1/2, and JNK [27, 28, 29, 30, 31, 32]. Activation of Akt and ERK1/2 suppresses pro‐apoptotic proteins such as Bim, Bad, and Bax, while enhancing the activity of Bcl‐xL/Bcl‐2, thereby promoting cell survival. These same pathways, along with JNK, upregulate matrix metalloproteinase 7 (MMP‐7), thereby increasing primary tumor cell invasion and migration. MMP‐7 expression can additionally be induced through SGK3/FOXO3 and p38 signaling. The SGK3/FOXO3 axis also downregulates DKK1, further supporting migratory potential. Beyond this, mesothelin overexpression broadly activates p38, NF‐κB, and STAT3 pathways. NF‐κB signaling enhances IL‐6 expression, reinforcing survival and proliferation. IL‐6 production is further enhanced by TNF‐α, and the two act together to stimulate cell proliferation and S‐phase entry under low serum conditions. Elevated IL‐6 levels also activate STAT3, which increases expression of the Cyclin E/CDK2 complex and accelerates the G1–S phase transition. NF‐κB and STAT3 signaling enhance the expression of anti‐apoptotic proteins such as Bcl‐xl, Bcl‐2, and Mcl‐1, thereby inhibiting apoptosis. MSLN further protects cancer cells from drug‐induced cell death by activating Akt phosphorylation through PI3K or engaging the MAPK/ERK pathway. Together, these pathways upregulate anti‐apoptotic genes, including Bcl‐2 and Mcl‐1, while repressing pro‐apoptotic factors such as Bad and Bax. The diagrams in panels (A), (B) and (C) were independently generated by the authors using BioRender based on information from the relevant literature [13, 21, 25, 26, 27, 28, 29, 30, 31, 32] and were used to generate an original figure. The three‐dimensional visualization of the molecule in (B) was done using the free version of the BIOVIA Discovery Studio Visualizer (Dassault Systèmes) (https://www.3ds.com/products/biovia/discovery‐studio/visualization).

The human *MSLN* gene is mapped to chromosome 16p13.3 [21]. It encodes a 71‐kDa precursor protein that undergoes proteolytic processing to yield two products: a 31‐kDa soluble fragment termed megakaryocyte potentiating factor (MPF) and a 40‐kDa membrane‐bound MSLN, which is tethered to the cell surface via a glycosyl‐phosphatidylinositol (GPI) anchor [16] (Figure 2A,B). These mature MSLN may also be released as “soluble MSLN‐related peptide” (SMRP) through proteolytic cleavage by enzymes such as “tumor necrosis factor‐α‐converting enzyme” (TACE/ADAM17) [13, 22]. Earlier, Dangaj et al. also reported that phospholipase D can cleave the MSLN GPI anchor, releasing soluble MSLN, which remains anchored via the GPI anchor [23]. The mannose portion of the GPI anchor may bind to the CD206 receptor on macrophages, potentially conferring immune‐modulating properties to MSLN [23]. MSLN mediates cell adhesion through its ability to bind the “tumor‐associated antigen 125” (CA125, also known as MUC16) [21]. Truncation and alanine‐scanning mutagenesis experiments have localized the CA125 binding site to a 64‐residue segment at the N‐terminal region of MSLN. MSLN‐expressing cells reside in the mesothelial layer lining of parenchymal organs and serosal cavities in a dormant state and do not proliferate until injured or stressed [24]. Under stress, MSLN forms a complex with CA125 and Thy1, influencing fibroblast activation and proliferation. Furthermore, MSLN's interaction with CA125 is influenced by its glycosylation state. CAR‐T cells targeting either the glycosylated C‐terminal region (487–598) or the N‐terminal region (296–390) exhibit differing levels of tumor cell killing. The observed differences could be due to varying accessibility of epitopes in the context of antigen shedding.

### 2.2 Signal Pathways

MSLN, whether in its soluble form or on the cell surface, activates several key signaling pathways, including NF‐κB, IL‐6/sIL‐6R, signal transducer and activator of transcription 3 (STAT3), Akt, extracellular signal‐regulated kinase (ERK)1/2, and JNK [27, 28, 29, 30, 31] (Figure 2C); thereby promoting epithelial–mesenchymal transition (EMT) and angiogenesis. NF‐κB proteins are transcription factors activated by inflammation and other stressors [32]. After activation, NF‐κB can induce IL‐6 expression. In addition, TNF‐α increases IL‐6 expression, promoting cell growth and progression through the S phase at low serum concentrations. Moreover, increased IL‐6 levels partially activate STAT3 and increase Mcl‐1 levels. These results indicate the existence of an “MSLN–NF‐κB–IL‐6–STAT3–Mcl‐1” survival pathway [32]. Akt may indirectly contribute to this axis through NF‐κB, forming a unique “MSLN–Akt–NF‐κB–IL‐6–Mcl‐1” survival mechanism in pancreatic adenocarcinoma (PDAC) cells. Inhibition of NF‐κB with wedelolactone reduces IL‐6 levels in “MIA‐MSLN” cells, and silencing IL‐6 decreases cell proliferation, slows the cell cycle, and induces apoptosis, accompanied by reduced c‐myc and Bcl‐2 expression [33]. Treatment with D3 Nb in MSLN‐overexpressing AsPC‐1 and Capan‐2 cells has been shown to reduce phospho‐AKT and phospho‐NF‐κB levels, and to significantly downregulate the EMT‐related genes twist‐related protein 1 (TWIST1)_and fibronectin 1 (FN1) at both the mRNA and protein levels [34]. These findings indicate that D3 Nb may inhibit EMT by blocking the AKT/NF‐κB pathway.

MSLN overexpression can also activate p38 [28, 29]. In MUC16‐expressing PDAC cells, MSLN is reported to promote MMP‐7 upregulation through a p38 MAPK‐dependent pathway. Suppression of MMP‐7 or inhibition of p38 activity has been reported to eliminate MSLN‐driven pancreatic cancer cell motility and invasion [35]. MSLN also drives cancer cell invasion and anoikis resistance via ERK1/2 signaling [36]. In MDA‐MB‐231 cells, MSLN overexpression influences ERK1/2 phosphorylation, which suppresses the accumulation of the proapoptotic protein Bim [37]. Bim commonly promotes apoptosis by neutralizing antiapoptotic proteins, including Bcl‐XL and Bcl‐2. However, ERK1/2‐mediated phosphorylation targets Bim for proteasomal degradation, thereby reducing its proapoptotic activity. This enables MSLN‐expressing cells to survive and proliferate under anchorage‐independent conditions, thereby contributing to their malignant and metastatic potential. Inhibition of ERK signaling with U0126 restores Bim levels and sensitizes cells to anoikis, confirming ERK‐dependent regulation.

In brief, *MSLN* is not simply a structural scaffold but an active oncogene that reconfigures cellular signaling pathways to facilitate invasion, metastasis, and evasion from programmed cell death. By maintaining hyperactivated signaling pathways, namely, NF‐κB, PI3K/Akt, and MAPK/ERK, *MSLN* confers a clear survival advantage to cancer cells. It is important to appreciate the biological basis for the wide array of *MSLN* expression profiles observed across solid tumors. Determining the expression pattern of *MSLN* across cancer subtypes is a key step toward establishing its role as a biomarker and informing the selection of immunotherapy strategies.

## 3 Pan‐Cancer Expression Landscape of MSLN and Its Biomarker Potential

**FIGURE 3:**
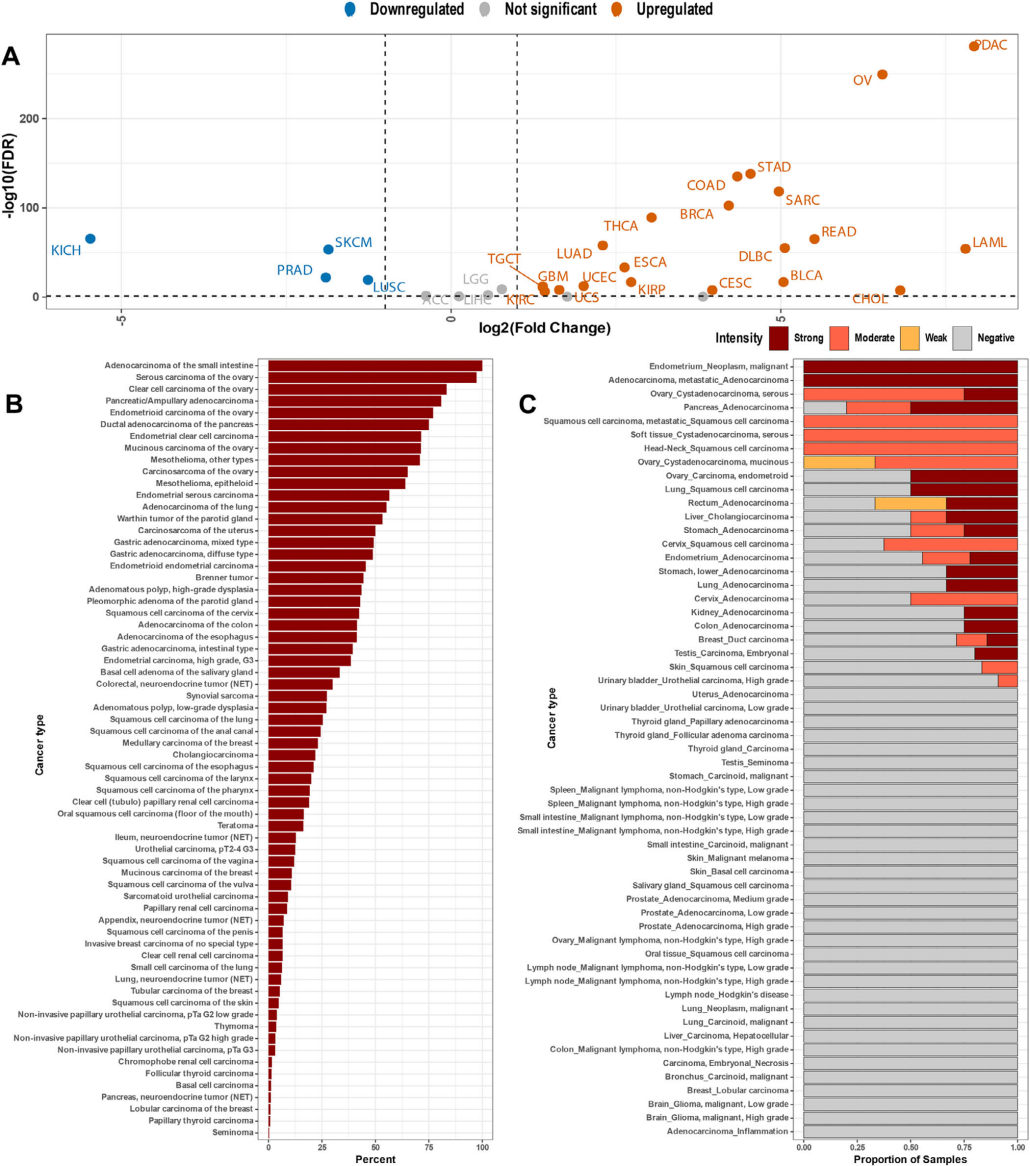
Pan‐Cancer MSLN Gene Expression Profile and Biomarker Potential. (A) Volcano plot showing differential expression of the *MSLN* gene across various cancer types, including bladder urothelial carcinoma (BLCA), breast invasive carcinoma (BRCA), cervical squamous cell carcinoma and endocervical adenocarcinoma (CESC), cholangiocarcinoma (CHOL), colon adenocarcinoma (COAD), lymphoid neoplasm diffuse large B‐cell lymphoma (DLBC), esophageal carcinoma (ESCA), glioblastoma multiforme (GBM), kidney renal clear cell carcinoma (KIRC), kidney renal papillary cell carcinoma (KIRP), acute myeloid leukemia (LAML), lung adenocarcinoma (LUAD), ovarian serous cystadenocarcinoma (OV), pancreatic adenocarcinoma (PDAC), rectum adenocarcinoma (READ), sarcoma (SARC), stomach adenocarcinoma (STAD), testicular germ cell tumors (TGCT), thyroid carcinoma (THCA), uterine corpus endometrial carcinoma (UCEC), uterine carcinosarcoma (UCS), kidney chromophobe (KICH), lung squamous cell carcinoma (LUSC), prostate adenocarcinoma (PRAD), and skin cutaneous melanoma (SKCM) in the GEPIA database. The x‐axis represents the log2 fold change in expression, whereas the y‐axis shows the ‐log10 of the FDR. Red dots indicate up‐regulated, blue dots represent downregulated, and gray dots signify nonsignificant differences in the expression profile of MSLN across different cancer types. (B) & (C) Bar graph displaying varying levels of MSLN expression intensity across different cancer types from Weidemann et al. [59] and Human Protein Atlas [57]. (B) This is a representative immunohistochemistry dataset from a study conducted by Weidemann et al. [59]. The x‐axis represents the percentage value, whereas the y‐axis shows the names of different cancer types. MSLN expression intensity values are represented using colors: dark red (strong intensity), red (moderate intensity), orange (weak intensity), and gray (negative). (C) This is a representative dataset from immunohistochemistry tissue microarray data from the Human Protein Atlas [57]. The x‐axis shows the proportion of samples, and the y‐axis indicates cancer types. MSLN intensity values are colored identically to those in panel B. This figure highlights differential expression of MSLN across cancer types at both the transcriptomic and proteomic levels, indicating its potential as a biomarker in certain cancers. Panels (B) and (C) were independently generated by the authors using data from Weidemann et al. [59] and the Human Protein Atlas [57] (https://www.proteinatlas.org/about/licence), respectively; both sources are used under the Creative Commons Attribution 4.0 International License (CC BY 4.0). All figures were generated using the ggplot2 package in R (version 4.3.2)

Given its role as an active oncogenic driver, the clinical importance of MSLN lies in its differential expression between malignant and normal tissues. In this section, we describe the expression pattern of MSLN across various cancers at both the transcriptomic and proteomic levels to demonstrate its suitability as a biomarker. *MSLN* is expressed at low levels in normal tissues but is significantly overexpressed in numerous tumor types, including ovarian cancers [38, 39, 40, 41], MESOs [41], malignant pleural mesothelioma (MPM) [42], colorectal cancer (CRC) [43], gastric cancer (GC) [44, 45, 46], and PDAC [39, 47, 48, 49]. Nonetheless, as mRNA levels may not necessarily reflect protein levels [50], we also explored publicly available gene and protein databases to identify cancers with a consistent signature. To understand the gene expression pattern, we utilized the Gene Expression Profiling Interactive Analysis (GEPIA) database [51, 52] and found that *MSLN* is significantly upregulated (Log_2_FC > 1 and FDR < 0.05) in 21 cancer types (Figure 3A), namely, bladder urothelial carcinoma, breast invasive carcinoma, cervical squamous cell carcinoma and endocervical adenocarcinoma, cholangiocarcinoma, colon adenocarcinoma, lymphoid neoplasm diffuse large B‐cell lymphoma, esophageal carcinoma, glioblastoma multiforme (GBM), kidney renal clear cell carcinoma, kidney renal papillary cell carcinoma (KIRP), acute myeloid leukemia (AML), lung adenocarcinoma (LUAD), ovarian serous cystadenocarcinoma (OV), PDAC, rectum adenocarcinoma, sarcoma (SARC), stomach adenocarcinoma (STAD), testicular germ cell tumors, thyroid carcinoma, uterine corpus endometrial carcinoma, and uterine carcinosarcoma—while it is significantly downregulated (Log_2_FC < −1 and FDR < 0.05) in four specific cancers: kidney chromophobe, lung squamous cell carcinoma (LUSC), prostate adenocarcinoma, and skin cutaneous melanoma. Since targeted therapies depend on high surface antigen expression [53], this review focuses primarily on the clinical implications of MSLN in cancer cells that overexpress it (Table 1). It is worth noting that there were no MESO gene expression datasets in the GEPIA database. Nevertheless, because MSLN protein overexpression occurs in 80–90% of MESOs [54, 55, 56], its clinical significance and potential as a therapeutic target have also been discussed. To understand the proteomic signature of MSLN, we also explored and analyzed immunohistochemistry (IHC) tissue microarray data from the Human Protein Atlas (HPA) [57] using the HPAanalyze [58] R package, as well as data published by Weidemann et al. [59]. The integration of transcriptomics with proteomics revealed that MSLN is upregulated mainly in cancer‐like, ovarian, pancreatic, gastric, colorectal cancers, and LUAD (Figure 3B,C).

**TABLE 1:** MSLN expression and clinical relevance in different cancer types.

Cancer type	MSLN expression level	Prevalence (%)	Clinical relevance	References
Ovarian carcinomas	Upregulated	50%		[60]
Ovarian carcinomas	Diffusion		Prolonged survival in some patients	[60]
Pancreatic carcinomas	Upregulated	50%		[60]
Ovarian carcinomas	Upregulated	71%		[61]
Ovarian carcinomas	Upregulated	67%		[62]
Ovarian carcinomas	High	68.8%	Poor prognosis	[63]
Ovarian carcinomas	Moderate	—	Tumor heterogeneity	[63]
Ovarian carcinomas	High		Poor response to chemotherapy and lower survival	[64]
Lung cancer	Positive	38–69%		[65, 66, 67]
Pancreatic carcinomas	Positive	38–69%		[9, 68]
Gastric cancer	Positive	21–78%	Poor outcomes	[69, 70, 71]
Gastric cancer	High		Poor patient prognosis	[14]
Gastric cancer	High	30%		[14]
Gastric cancer	High			[14]
Gastric cancer	High	50–55%	Aggressive tumor features, a significantly higher 5‐year survival rate	[72]
Gastric cancer	High	49.7%	Poor prognosis	[71]
Pancreatic cancer	High	90%		[10]
Pancreatic cancer	High	91.7%		[73]
Pancreatic cancer	High	62%		[74]
Colorectal cancers	Positive		Proliferation of colon cancer cells	[75]
Colorectal cancers	Positive		Poor survival	[76]
Colorectal cancers	Positive	17%	Nonluminal expression profile is involved in the aggressiveness of the tumors	[77]
Colorectal cancers	High	10%	Worse prognosis	[78]
Mesotheliomas	High	80–90%	Poor clinical outcomes	[79, 80, 81, 82, 83]
Triple‐negative breast cancer	High	34%	Poor survival	[84]
Triple‐negative breast cancer	High		Poor clinical outcomes	[79, 80, 81, 82, 83]
Triple‐negative breast cancer	High	42.3%	No correlation with prognosis	[85]
Triple‐negative breast cancer	High		No impact on survival	[86]
Lung adenocarcinoma	High	53%	Poor survival	[87]
Lung adenocarcinoma	High		Poor clinical outcome	[88, 89, 90]
Lung adenocarcinoma	High	20%	poor overall survival	[91]
Acute myeloid leukemia	High	36%		[92, 93, 94]

### 3.1 Ovarian Cancer

Using several Serial Analysis of Gene Expression (SAGE) libraries from cancer and noncancerous tissue samples, earlier researchers found that *MSLN* is selectively upregulated in 50% of patients with ovarian cancer and pancreatic carcinomas, a rate considerably higher than in normal tissues [60]. An extensive cohort study using immunohistochemical analysis suggested that MSLN was expressed in approximately 71% of ovarian cancer cases, indicating that MSLN expression is common in epithelial ovarian cancer [61]. Using a sandwich enzyme‐linked immunosorbent assay (ELISA), one study demonstrated that serum MSLN was elevated in 67% of ovarian cancer patients, supporting its potential as a biomarker for MSLN‐expressing tumors [62]. High levels of MSLN were also reported in the 68.8% of epithelial ovarian cancer cases and were significantly linked to poor prognosis. MSLN levels in serum, measured by ELISA, showed a moderate correlation with tumor expression and suggested tumor heterogeneity [63]. However, despite controversies surrounding its clinical utility as a predictive marker, increased levels of MSLN in ovarian cancer patients have been consistently linked to worse prognosis, especially for high‐grade serous tumors [62]. High MSLN expression links to a poor response to chemotherapy and lower survival in advanced disease, regardless of cytoreductive surgical intervention [64]. However, the predictive significance of MSLN has not received universal endorsement, as several studies have reported no significant association [40]. In contrast, diffuse expression has been associated with prolonged survival in some patients [60].

### 3.2 Pancreatic Cancer

To date, numerous studies have examined the potential usefulness of MSLN in both cytology and histology of pancreatic tissues. In all these studies, MSLN has shown sensitivity, specificity, and diagnostic accuracy of 75, 80, and 77%, respectively, in detecting PDAC. Because of its clinical value, the Papanicolaou Society of Cytopathology included MSLN staining as an adjunctive tool for PDAC diagnosis in 2014 [73, 95]. The initial report on MSLN in PDAC was published in 2001, in which Argani et al. showed transcript expression in 100% of primary tissues (*n* = 4) by in situ hybridization and in 90% of cell lines examined (18 out of 20) by reverse transcription polymerase chain reaction (RT‐PCR) [10]. To validate MSLN as an antitumor target, a large‐scale study examined the immunohistochemical expression of MSLN in various solid cancers using two different mouse monoclonal antibodies (mAbs). For example, in PDAC cancer, the expression of MSLN was observed to be very high in both 5B2 and MN‐1 clones, at 71 and 87%, respectively [69]. The differences observed in the presence of MSLN are likely due to antibodies used for detection, which target different epitopes and exhibit different sensitivities [73].

In another study, the scientists [73] conducted the first clinical assessment of MSLN expression in PDAC using two antibodies, EPR4509 and EPR19025‐42. Both antibodies showed excellent signal quality and total agreement on diagnostic results for each patient sample. Moreover, MSLN expression levels were increased in 91.7% of PDAC samples, with no expression detected in adjacent paraneoplastic tissues. In particular, it was assumed that the significantly higher percentage of MSLN‐positive cases in PDAC than in other solid tumors is due to specific epigenetic modifications, such as differential methylation of MSLN. In a study of 65 cases of pancreatic neoplasms, Jhala et al. found that the MSLN positivity rate in adenocarcinomas was 62%, compared with 0% in reactive chronic pancreatitis [96]. Such a large difference indicates that MSLN immunohistochemical staining is highly useful for distinguishing pancreatic ductal adenocarcinoma from chronic inflammation, thereby suggesting the presence of malignant ductal epithelial cells [74]. This has led to a growing focus on assessing MSLN's potential as a biomarker for the clinical detection of PDAC. For example, in one study, MSLN f expression in pure pancreatic juice was detected in 52, 45, and 14% of patients with PDAC, intraductal papillary mucinous neoplasms (IPMNs), and chronic pancreatitis, respectively [97]. The method showed a sensitivity of 52% and a specificity of 86% in detecting invasive PDAC. Further studies investigating precursor lesions showed that MSLN was expressed in 21 out of 37 IPMNs, and luminal membrane localization was a robust predictor of recurrence. In general, the available literature indicates that MSLN is highly abundant in invasive PDAC and in high‐risk preinvasive cases; however, it is absent in normal pancreatic tissue, thereby highlighting its substantial clinical value as both a diagnostic and prognostic biomarker.

### 3.3 Gastric Cancer

The recent study by Saha et al. [14] provided a complete characterization of *MSLN* expression in GC through both bioinformatics and experimental analyses. The bioinformatics study demonstrated a substantial increase in *MSLN* expression in GC and in nine other cancers. Moreover, an in‐depth evaluation of 375 GC cases and 32 controls from the TCGA‐STAD cohort demonstrated a substantial overexpression of *MSLN* transcripts. This finding was later corroborated by RT‐qPCR experiments on Eastern Indian GC cases, which showed that MSLN transcript abundance was significantly higher in tumors than in adjacent nontumor tissues. Further proteomic analysis also confirmed the overexpression of MSLN in 30% of GC cases. To investigate the potential clinical use of the biomarker, the authors further showed that plasma MSLN concentration was significantly higher in GC cases than in controls (*p* < 0.001). Receiver operating characteristic analysis showed an area under the curve of 0.67. This suggests that plasma MSLN may have modest utility in distinguishing patients with GC.

Another more extensive immunohistochemical study involving 958 patients with advanced GC showed that high levels of MSLN were a clear indicator of poor prognosis [71]. They noted that high MSLN levels were present in 49.7% of patients and correlated significantly with advanced T stages (*p* = 0.021). In addition, multivariate analysis indicated that high MSLN was an independent predictor of poor overall survival (OS), poor recurrence‐free survival, and peritoneal metastasis. The predictive value of high MSLN was especially pronounced among patients with Stage III and diffuse/mixed GC. Another study reported that increased MSLN levels indicate a poor prognosis for GC and are a strong risk indicator for peritoneal recurrence. More specifically, MSLN, together with Lauren classification and tumor staging, represents a significant predictor, particularly for peritoneal recurrence in patients at Stage III [71].

However, a retrospective cohort of 212 patients undergoing R0 gastrectomy also underscores the complex nature of MSLN's prognostic value in GC, as assessed by IHC [72]. In their study, the authors reported that 59% of the tumors studied expressed MSLN (defined as >50% membrane staining). Of note, MSLN expression was associated with aggressive tumor features such as deeper tumor invasion and lymph node involvement (*p* < 0.05). Notably, despite its association with unfavorable tumor features, MSLN expression was found to predict a survival advantage in patients with late‐stage disease. Indeed, when multivariate analysis was performed in a subgroup of 117 patients diagnosed with advanced GC, a significantly higher 5‐year survival rate (55%) was identified in patients with positive MSLN expression compared with patients without MSLN.

### 3.4 Colorectal Cancers

MSLN expression is also an important predictor for patients diagnosed with CRCs. In a large‐scale IHC study of 270 primary tumors and 44 metastases from CRC, diffuse luminal or membranous MSLN was an independent risk factor for poor OS [75]. It is essential to note that MSLN expression in primary CRC tumors was positively associated with expression in metastatic sites. Furthermore, the in vitro findings that show MSLN promotes the proliferation of colon cancer cells indicate that MSLN plays an important role in both prognosis and therapy of metastatic CRC. Localized CRC patients with MSLN‐positive expression in their primary tumors were found to be very predictive markers of tumor recurrence [76]. MSLN‐positive expression demonstrated a high sensitivity of 93% for predicting future peritoneal metastases. Furthermore, MSLN was associated with poor survival, underscoring its prognostic significance. In a study involving 530 patients with Stage II and III colon cancers, researchers found that 17% of patients had MSLN‐positive tumors [77]. Of these MSLN‐positive tumors, most (62%) had a nonluminal expression profile, which independently correlated with a significantly decreased survival time from the primary cancer in both Stages II. The authors suggest that the nonluminal expression profile is associated with tumor aggressiveness due to direct contact with stromal cells, whereas luminal expression (38%) was not associated with clinical outcome. Another study on 640 patients of CRC [78] found that ∼10% of them have overexpression of MSLN similar to that observed in ovarian cancer patients. Interestingly, such high expression is often associated with mutations in KRAS or BRAF and a poor patient outcome.

### 3.5 Mesotheliomas

MESO is a rare form of aggressive cancer. Because there are few treatment methods available and the condition develops rapidly, the prognosis for those suffering from MESO is usually poor. Because of elevated MSLN levels in MESO patients, MSLN has become a major focus of MESO research [8]. MSLN protein levels are markedly elevated in the epithelial type of MPM, the most prevalent subtype (80% of cases) [54]. Notably, although less than 3% of epithelial MPM patients develop distant metastasis, most of them are not candidates for radical surgery because of the advanced stage at which their tumors are diagnosed and the degree of tumor invasion into other tissues in the body. They usually have T3 tumors that have aggressively invaded the endothoracic fascia, mediastinal fat, chest wall, or pericardium, unlike T2 tumors, which do not show evidence of invasion. The invasion process is partly facilitated by matrix metalloproteinases, which degrade the basement membrane, enabling tumor cells to invade. In addition, asbestos, the causative agent in most cases of MESO, also induces expression of both MSLN and matrix metalloproteinase‐9 (MMP‐9). Another study with 82 cases of MPM [98] reported MSLN in more than 64% of cases. 49% of those cases showed high levels of the marker, suggesting a potential role in predicting treatment effectiveness and survival. High MSLN levels were associated with diffuse D2‐40 positivity, likely because both markers are located in the cell membrane. On the other hand, nuclear expression of WT‐1 and BAP‐1 had no significant association with MSLN positivity. Low MSLN levels were positively associated with tumor necrosis and nuclear grade. This result is consistent with findings from Bharadwaj et al. [32], who showed that high MSLN levels enhance resistance to TNF‐α‐induced apoptosis through the Akt/phosphoinositide 3‐kinase/nuclear factor kappa B signaling pathway.

### 3.6 Other Cancer Types

Overexpression of MSLN in breast cancer is mostly limited to the Triple‐negative breast cancer (TNBC) subtype and promotes increased cell proliferation, a proinflammatory microenvironment, and ultimately poor clinical outcomes [79, 80, 81, 82, 83, 99]. Another study reported that MSLN expression in TNBC was significantly higher than in non‐TNBC tumors [83]. The overexpression of MSLN significantly elevated the levels and activity of ERK1/2 and MMP9, thereby contributing to greater invasiveness and angiogenesis in MCF‐7 breast cancer cells [100]. Recently, RNA sequencing of the 4T1‐HM3 and 4T1‐Pri metastatic cancer cell lines demonstrated upregulation of the MSLN gene in metastatic TNBC, which was further confirmed by quantitative real‐time PCR (qRT‐PCR), Western blotting, and IHC [101]. Overexpression of MSLN is significantly linked to metastasis to the liver rather than metastasis to other locations. In addition, MSLN binds to EGFR, activating the ERK1/2 signaling pathway and thereby increasing TNBC cell survival and proliferation during metastasis. MSLN expression was detected in 42.3% of TNBC patients and was positively correlated with cancer grade. Nevertheless, no correlation with prognosis was reported for MSLN expression [85].

MSLN is significantly upregulated in non‐small cell lung cancer (NSCLC) tissues and lung carcinoma, where its function is linked to EMT and stemness characteristics [88, 89]. MSLN expression is found to be high in the serum as well as tumors of NSCLC patients with brain metastasis (BM) and is linked to poor clinical outcome. From a functional perspective, MSLN promotes brain metastatic behavior in NSCLC cells, particularly by facilitating infiltration across the blood–brain barrier (BBB). This occurs because MSLN promotes MET expression and activation via JNK signaling pathways, leading to BBB disruption and tumor cell infiltration [90]. In another report, MSLN was expressed in 69% of early‐stage LUADs, with high‐level expression in about 20% of cases. High MSLN expression was associated with poor overall and recurrence‐free survival in a multivariate analysis. In an orthotopic and metastasis mouse model system, both human and murine lung ADCs overexpressing MSLN showed increased proliferation, accelerated tumor progression, and decreased survival [91].

MSLN is also expressed in AML, but its role in AML pathogenesis remains unclear. It is important to note that MSLN is expressed in about a third of childhood AML patients, particularly those with KMT2A‐rearranged AML and core binding factor (CBF) AML [102]. Studies have demonstrated low MSLN expression in healthy bone marrow but high expression in myeloid cells in some cases of AML. It is worth noting that MSLN expression levels return to normal when the disease remits [93]. This is evidenced by Meshinchi et al., who performed a transcriptomic analysis with *n* = 2051. In addition, circulating MSLN levels have been detected in the serum of AML patients [94]. MSLN expression, detectable at both the transcript and protein levels, is an independent risk factor for extramedullary disease in AML. Multivariate analyses demonstrated that transcript‐level MSLN positivity and MSLN expression on leukemic blasts by flow cytometry are each independently associated with an increased risk of extramedullary disease. Mechanistically, MSLN overexpression promotes AML cell proliferation, migration, and invasion, and facilitates cell–cell adhesion through its interaction with MUC16, thereby contributing to extramedullary dissemination [103]. In addition, MSLN protein has been identified as an emerging biomarker and new immunotherapy target for human GBM. MSLN has been shown to elicit potent cellular and humoral immune responses in cancer patients and is detectable in blood serum [104]. Nevertheless, like its tissue expression in other glands such as the thyroid and the prostate, the current body of knowledge regarding GBM is very limited. Further investigations are needed to fully establish its biological significance and its potential clinical use as both a marker and a treatment target.

### 3.7 MSLN is Coexpressed With CA125

It is well established that MSLN mediates both cell–cell and cell–matrix adhesion by binding to CA125, thereby aiding the peritoneal spread of cancers overexpressing MSLN [13, 105]. In one study, MSLN expression along with CA125 coexpression was associated with poor prognosis in pancreatic cancer patients [106]. In a cohort of 478 breast cancer cases, a significant positive association was observed between MSLN expression and its receptor, CA125 (*p* = 0.0004). Coexpression of both biomarkers was observed in 10% of patients and was significantly associated with poor relapse‐free survival (*p* = 0.0001) [107]. It was also shown that this axis mediates heterotypic cell–cell adhesion. This refers to the ability of the human ovarian cancer cell line OVCAR3, which expresses CA125, to adhere to endothelial‐like cells expressing MSLN [108]. Building on these results, Gubbels et al. revealed the high specificity of MSLN's binding to CA125, with this interaction being dependent on N‐linked glycans. Most importantly, this dependence on glycans ensures that CA125‐positive ovarian tumor cells can attach specifically to the MSLN‐positive peritoneal layer [109]. In support of the significance of the MSLN–CA125 complex in the adhesion process, Bruney et al. reported that when MT1–MMP is overexpressed in human ovarian carcinoma cells (OVCA433‐MT), the surface expression of CA125/MUC16 on the cell membrane was reduced. In addition, overexpression of MT1–MMP increases shedding of the CA125 ectodomain, thereby eliminating its cell‐surface expression. As a result, these cells showed reduced binding to human mesothelial cells (LP9) and peritoneal explants [110]. Once ovarian cancer cells have attached to the peritoneal mesothelium, the coexpression of MSLN and CA125 proteins recruits additional ovarian cancer cells that are shed from the initial tumor site [28]. Thus, secondary tumor burden may include excessive cell proliferation and adhesion to circulating single‐ or multicellular clusters within the ascitic peritoneum [109]. In contrast, the physiological role of the MSLN protein in tumor development remains unknown [111], but determining the importance of CA125–MSLN interactions may help develop new treatment options for ovarian peritoneal metastasis.

In short, MSLN is an excellent biomarker candidate, positively correlated with poor prognosis across a wide variety of cancers. In particular, MSLN expression is elevated in several types of cancer, such as ovarian, pancreatic, and gastric adenocarcinomas and MESO, thus justifying its application both in diagnostic and prognostic settings. Furthermore, the functional association between MSLN and CA125 underscores MSLN's contribution to peritoneal metastasis and progression. Despite its obvious relevance in clinical practice, using MSLN as a biomarker and treatment target poses challenges due to its inter‐ and intratumoral heterogeneity, meaning that MSLN expression varies significantly not only between cancer types but also within a single type. Therefore, there must be other mechanisms of MSLN regulation beyond those revealed by transcriptomic and proteomic analyses. To identify these alternative pathways, it is important to investigate MSLN dysregulation at the molecular level. The discussion below focuses on the epigenetic mechanisms that regulate MSLN expression. In particular, this review focuses on DNA methylation, which is crucial for understanding the molecular mechanisms behind the dysregulated MSLN expression.

### 3.8 Association Between MSLN Expression and OS Across Cancer Types

The significance of MSLN prognosis was examined further with regard to OS on the GEPIA platform. Kaplan–Meier survival analysis demonstrated the influence of MSLN prognosis on OS in different cancers, where high levels of MSLN were significantly associated with good OS in HNSC and KIRP (Figures S1–S8). Meanwhile, high MSLN expression was associated with poor OS in brain lower‐grade glioma and LUAD. Despite a statistical difference in OV, the median difference in OS was rather small between high and low MSLN expression. Overall, it can be concluded that the prognostic significance of MSLN is cancer‐type dependent.

## 4 Pan‐Cancer Epigenetic Landscape of MSLN

Although MSLN overexpression and its molecular associations (mainly CA125) are important factors in cancer progression, understanding how MSLN is regulated upstream to achieve this abnormal expression is equally important. There is increasing evidence that the regulation of MSLN expression in different cancers is epigenetic, particularly through DNA methylation. Epigenetic modifications such as DNA methylation, phosphorylation, acetylation, and ubiquitylation, along with histone and chromatin remodeling, are heritable alterations in gene expression that do not involve any change in the primary DNA sequence. Among these, DNA methylation is the most widely researched epigenetic modification. DNA methylation occurs by the addition of a methyl group to the carbon‐5 position of cytosine (5mC). Dysfunctional DNA methylation has been implicated in many diseases, including cancers, metabolic diseases, heart diseases, infections, neurodegenerative disorders, and allergies, among others [112, 113]. Earlier studies have also reported that MSLN plays a vital role in epigenetic regulation and signaling pathways [114]. MSLN transcription is regulated by mechanisms such as promoter methylation and alternative splicing. Higher MSLN expression levels are associated with increased tumor invasiveness, even though this is not always due to binding to MUC16. Numerous studies have investigated the behavior of MSLN‐positive tumors in both in vitro and in vivo settings to better understand the implicated signaling pathways. Although there is no single primary mechanism of action, MSLN suppression is associated with reduced proliferation and tumor growth in vivo [115].

In earlier studies, SMRP levels have been used to examine MSLN expression in MPM tumors. This investigation has revealed that there are three methylated CpGs in the MSLN promoter, 214 bp upstream from the transcription start site. These methylation sites showed significantly higher methylation levels in normal pleural tissues than in tumor tissues (*p* < 6.0 × 10^−^^9^). As a result, MSLN promoter hypomethylation can be associated with MSLN overexpression in MPM. Also, tumors from SMRP‐negative patients had significantly higher methylation levels than those from SMRP‐positive patients [116]. Therefore, it can be concluded that methylation of MSLN is common in normal pleural tissues, whereas most MPM tumors exhibit demethylated MSLN promoters, leading to elevated MSLN levels. However, in a small subset of tumors, promoter methylation is retained, which may account for the decreased sensitivity of SMRP assays. In addition, histo‐epigenetic analysis has classified MSLN‐expressing tumors into distinct groups based on expression levels, with PDAC and MPM as examples [117].

An integrative analysis of gene expression and DNA methylation profiles in patients with matching RNAseq and methylation profiles (*N* = 246) showed a significant correlation between promoter‐region hypomethylation of MSLN and higher MSLN expression in AML [118]. In a separate study, the MSLN expression profile was determined by IHC, while methylation of 20 CpG sites in the MSLN gene promoter was examined in 118 lung samples [119]. These comprised 39 samples of malignant MESO (MM), 41 lung carcinomas, 26 non‐neoplastic lung lesions, and 12 normal lung samples. The methylation analysis was performed using a methylation‐sensitive single‐nucleotide primer extension assay. From the results obtained, it can be noted that MSLN expression was observed in the epithelioid type and the epithelial component of biphasic MM, whereas it was not seen in the sarcomatoid type and the sarcomatous component of biphasic MM. It is interesting to note that MM showed significant hypomethylation of the MSLN promoter, regardless of subtype, compared with other pulmonary lesions and normal lungs. These observations indicate that MSLN promoter hypomethylation may be specifically associated with MM, without any correlation with MSLN expression. Also, the lack of MSLN expression in the sarcomatoid subtype of MM might be due to an unknown posttranscriptional regulation mechanism. In addition, four of the 20 CpG sites analyzed showed greater specificity for hypomethylation in MM samples. However, few reports have examined DNA methylation associated with MSLN, underscoring the need for further studies. Moreover, there are also little data on other types of epigenetic modifications.

To summarize, epigenetic modifications, including DNA methylation, have been identified as major regulators of MSLN transcription. It is important to emphasize that epigenetic modifications are not static and exhibit significant plasticity, constantly changing in response to various selection pressures in the tumor environment. Due to the dynamic nature of epigenetic modifications, MSLN transcription levels can vary widely among patients and across cell types within a single tumor mass. Such basic epigenetic plasticity may serve as a primary source of MSLN variability, as described in detail in the following section.

## 5 Tumor Heterogeneity of MSLN

Tumor heterogeneity can be categorized into two types: intertumoral and intratumoral heterogeneity. Intertumoral heterogeneity refers to the biological differences among patients with the same histologically characterized cancer. Intratumoral heterogeneity refers to phenotypic and genotypic differences among subpopulations within a single tumor [120, 121]. Intratumor heterogeneity has been classified into two groups, namely, temporal and spatial heterogeneity. Temporal heterogeneity refers to the development of an individual tumor over time, driven by changes in its genetics and phenotype, thereby giving rise to unique subpopulations at various phases of cancer progression [121]. During tumor development, the changing microenvironment favors clones that can survive through angiogenesis and invasion [122]. However, spatial heterogeneity refers to the uneven distribution of various cancer cell types within the same body region or across different lesion sites in the same individual [123]. Importantly, this heterogeneous complexity is among the major factors limiting the effectiveness of therapies targeting MSLN. Since MSLN expression is subject to dynamic changes driven by epigenetic mechanisms, it is generally not uniformly distributed; hence, diagnostic screening and therapeutic interventions must account for both patient and tumor heterogeneity.

### 5.1 Interpatient Heterogeneity

Intertumor heterogeneity is a major obstacle to contemporary precision medicine approaches [121]. For instance, studies have shown that LUAD tumors exhibit intertumor genetic heterogeneity and pathway alterations [124]. In particular, the adenocarcinoma invasion subtype shows a remarkable number of mutations in genes associated with the mTOR and Hippo pathways. Specific mutations are associated with differential prognosis in LUAD patients, underscoring the importance of personalized treatments across subtypes [120]. Thus, by studying interpatient heterogeneity, we can gain deeper insight into tumor diversity and develop effective approaches to treating it. As shown in the transcriptomic dataset from GEPIA (Figure 3A), MSLN expression levels vary across cancer subtypes. For example, patients with ovarian and pancreatic cancers experience a significantly higher log fold‐change in MSLN expression compared with other cancer subtypes. At the protein level, MSLN expression is also heterogeneous across different cancer types (Figure 3B,C). For example, using immunohistochemical staining, we found that the majority (>98%) of patients with serous/clear cell carcinoma of the ovary, as well as with pancreatic ductal adenocarcinoma, have MSLN‐positive tumors. At the same time, in cases such as LUSC and medullary carcinoma of the breast, MSLN is detectable in only specific subpopulations of patients (∼10–40%) (Figure 3B). Specifically, although the majority of patients with endometrial and metastatic ovarian adenocarcinoma have the “strong” (dark red) level of MSLN expression, the PDAC patient group is quite diverse: some have strong staining, others have moderate MSLN levels, and yet another portion does not express MSLN protein (Figure 3C). Finally, in cases of lymphoma and glioma, none of the patients tested expressed MSLN. All this information shows that the “one‐size‐fits‐all” strategy would be inapplicable to MSLN‐targeted therapeutic approaches. Therefore, it is critical to develop appropriate methods for molecular screening of patient populations and to enroll only those with sufficient MSLN protein expression in clinical trials. There is also notable interindividual heterogeneity in MSLN expression across cancer types. For example, MSLN expression in pancreatic cancer may vary based on classical and basal‐like subtypes (Figure 3B).

These findings are consistent with previous research demonstrating variability in MSLN expression between patients [13, 43, 56, 125]. For example, the soluble form of MSLN is a distinct biomarker in MPM occurring in patients with asbestos exposure [126]. MSLN is highly expressed in epithelioid as compared with sarcomatoid MESOs [56, 69, 127]. Moreover, increased MSLN expression is observed in the consensus molecular subtype 1 (CMS1) and consensus molecular subtype 4 (CMS4) subtypes of CRC, which are associated with elevated mutation rates [43]. However, in a recent large‐scale transcriptomic analysis of 2051 pediatric and young adult patients with AML, it was reported that MSLN overexpression is not limited to solid tumors. According to the findings, MSLN was upregulated in about 36% of AML cases but absent in normal bone marrow cells. MSLN protein was also highly expressed in cases with chromosomal aberrations, including KMT2A rearrangements and inv(16)/t(16;16), as well as t(8;21) fusions (core‐binding factor fusions), and extramedullary disease [94, 128]. In addition, there is heterogeneity of MSLN expression in pancreatic cancer, which can be noted between the primary site and its metastasis. For instance, in one patient, the primary lesion had a stronger expression (3+) than the metastasis (2+) [129]. IHC of ovarian cancer demonstrated MSLN positivity in 2041 of 2342 (87%) evaluable tissue spots and 372 out of 392 (95%) interpretable tumor samples [130].

Of 109 TNBC patients, 34% were MSLN‐positive, and MSLN positivity had no effect on patient survival [84]. Of particular interest, MSLN overexpression has been reported in about 67% of patients with TNBC alone but is rare in ER+ and HER2+ tumors [81]. One study showed that MSLN expression is much more common in TNBC than in non‐TNBC (36% versus 16%; *p* = 0.0006) and is associated with reactivity to basal keratin antibodies but not with EGFR [83]. No differences in clinical–pathological parameters, such as race, size, grade, subtype, and lymphovascular invasion, as well as nodal involvement, were observed between patients with MSLN‐positive and MSLN‐negative TNBC; however, patients with MSLN‐positive tumors were older, experienced earlier dissemination, and had poorer overall and progression‐free survival (PFS). In another study, spatial transcriptomics of T1N3‐stage TNBC revealed that MSLN is highly expressed in this aggressive subtype of breast cancer [131]. 32.8% of total breast cancers and 57% of TNBC were found to exhibit high levels of MSLN, and this observation correlated significantly with the TNBC subgroup and lack of estrogen receptor, progesterone receptor, and HER2 expression [80].

Among 93 patients diagnosed with advanced LUAD, 53% were found to express MSLN, with high expression (>25% of tumor cells) noted in 24% of patients. Higher MSLN expression was significantly associated with poor survival (median survival 18.2 vs. 32.9 months; *p* = 0.014). In molecular studies, an association was found between MSLN and the presence of a KRAS mutation (*p* < 0.0001) and EGFR wild‐type status (*p* = 0.002) [87]. The high expression level of MSLN was also linked to male gender, smoking, and papillary‐predominant LUAD. It is important to mention that MSLN characterized a unique group of lung ADC patients with an unfavorable prognosis who had no other aggressive factors like VPI and lymphovascular invasion. Furthermore, MSLN expression proved to be an independent indicator of unfavorable prognosis regardless of smoking and KRAS mutation [91].

The importance of MSLN for prognosis prediction is especially evident in certain molecular subtypes of AML. For example, in the CBF‐abnormal subtype, the prevalence of MSLN overexpression reached 72% [92]. In survival analysis, the presence of MSLN was associated with an increased likelihood of relapse (51 vs. 32%) and decreased disease‐free survival (46 vs. 64%), suggesting that lower MSLN expression correlated with better patient prognosis [132]. In addition, the overexpression of MSLN was associated with a higher probability of developing extramedullary disease (specifically, the incidence rates were 27.8 and 18.8% for MSLN‐positive and MSLN‐negative patients, respectively. This association was especially evident for relapsed or refractory AML. MSLN is expressed in about 36% of AML patients but is low in healthy hematopoietic cells, making it an excellent candidate for developing therapeutic agents for AML [92].

### 5.2 Intratumoral Heterogeneity

**FIGURE 4:**
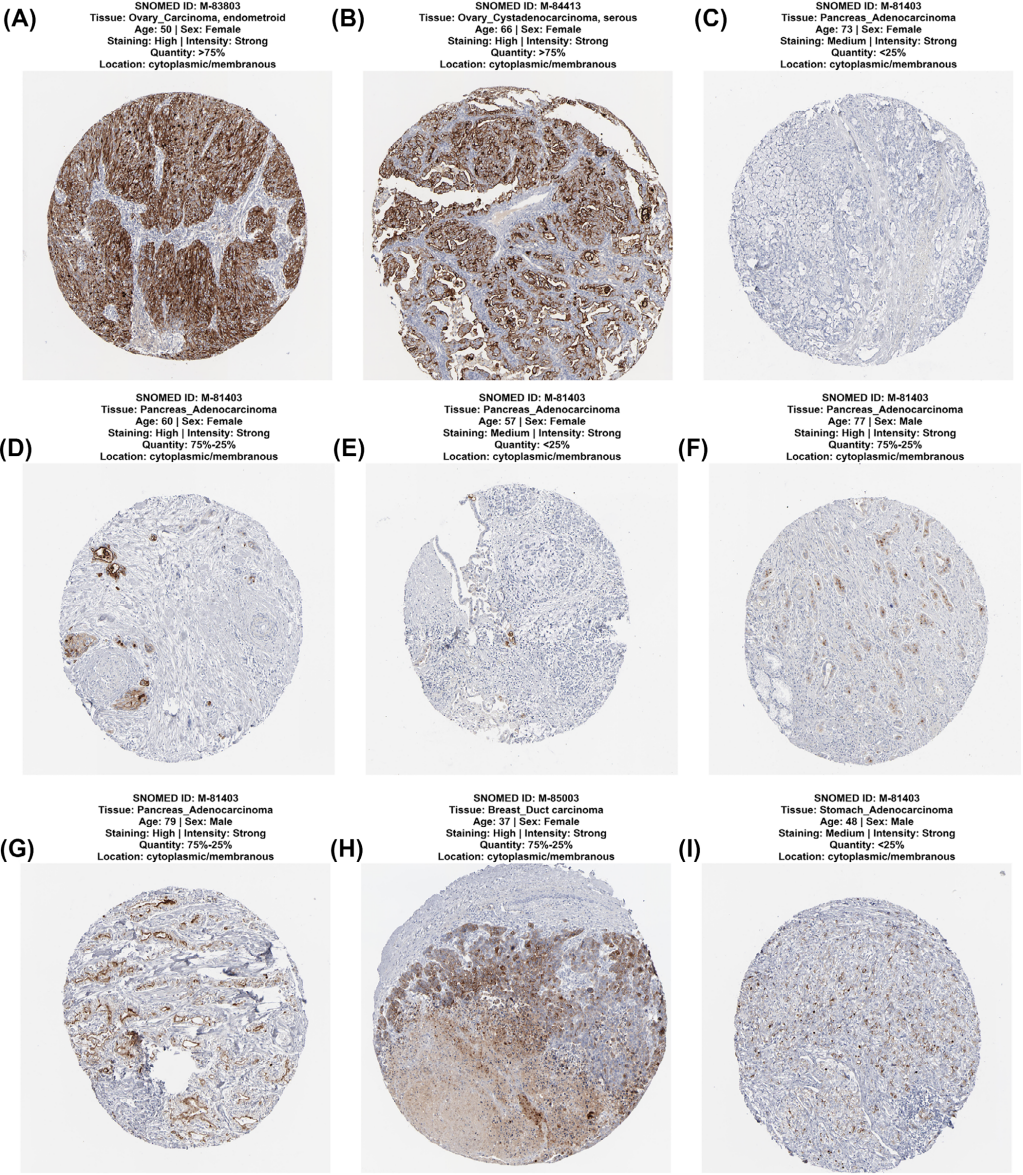
Immunohistochemical profiling reveals pronounced spatial and subcellular heterogeneity of MSLN expression across diverse solid tumors. (A) Ovary Carcinoma endometrioid; (B) Ovary Cystadenocarcinoma serous; (C‐G) Pancreas Adenocarcinoma; (H) Breast Duct carcinoma; (I) Stomach Adenocarcinoma. Tissue microarray cores adapted from the Human Protein Atlas demonstrate MSLN staining (brown) in various malignancies. Notably, while all presented tissue cores exhibit “Strong” staining intensity, the spatial distribution of the antigen varies drastically. Ovarian carcinomas (A and B) exhibit ubiquitous expression, with >75% of cells staining positive. Conversely, pancreatic (C‐G), breast (H), and stomach adenocarcinomas (I) routinely exhibit highly focal, patchy distribution, with antigen expression restricted to <25% or 25–75% of the malignant compartment, leaving substantial regions of the tumor entirely devoid of MSLN. Furthermore, all samples shown exhibit mixed “cytoplasmic/membranous” subcellular localization, highlighting the intracellular compartmentalization that limits the accessibility of targets for large macromolecular therapeutics. Images were generated using the HPAanalyze package [58] in R under the Creative Commons Attribution 4.0 International License.

**FIGURE 5:**
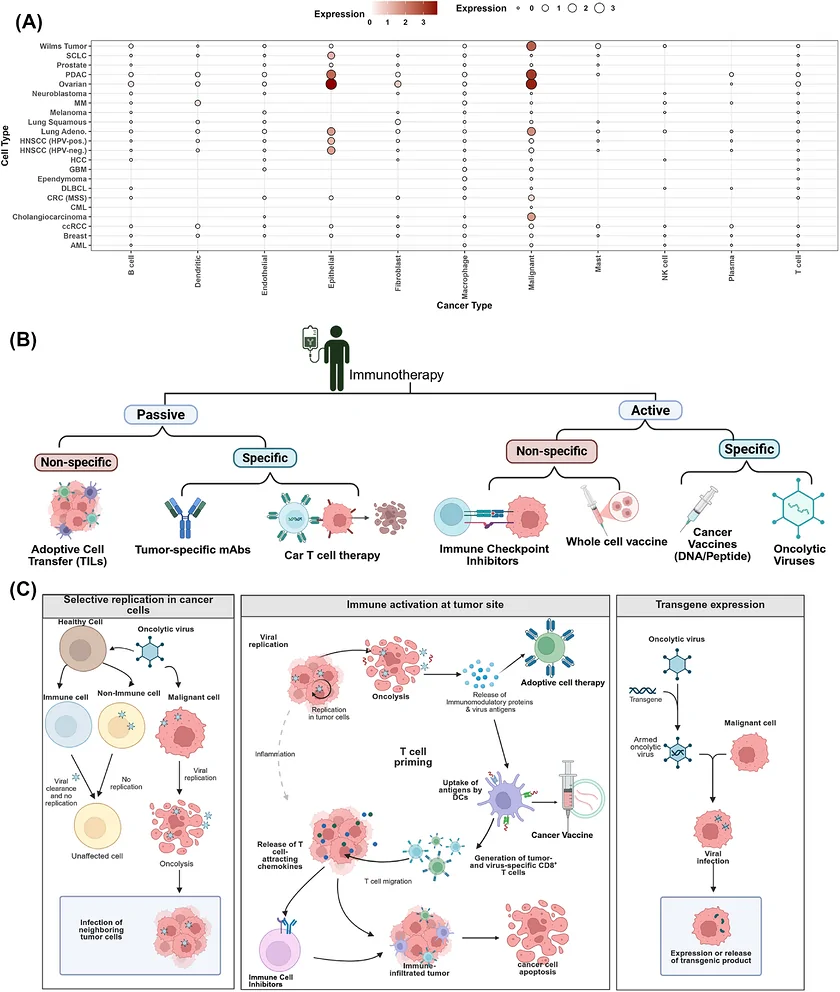
The distribution of mesothelin in single cells, and its potential for targeting advanced therapies. (A) A bubble plot of single‐cell RNA sequencing (scRNA‐seq) data shows MSLN expression across different cancer types within the tumor microenvironment [136]. The circle's size and color intensity (from light to dark red) both indicate the level of *MSLN* expression. MSLN is primarily found in malignant epithelial cells (such as those in PDAC, ovarian cancer, and lung adenocarcinoma) and is less abundant in immune cells. (B) Immunotherapeutic approaches are broadly categorized into Passive and Active therapies, which are further subdivided by targeting precision (Non‐specific vs. Specific) [143, 149, 150]. Passive immunotherapy involves the introduction of foreign immune substances, including non‐specific tumor‐infiltrating lymphocytes and specific substances such as monoclonal antibodies and chimeric antigen receptor T‐cell therapy. Active immunotherapy attempts to elicit an immune response within the patient's body; examples of non‐specific approaches include immune checkpoint inhibitors and whole‐cell vaccines, while specific approaches include targeted cancer vaccines (DNA/peptide vaccines) and oncolytic virus vaccines. (C) This schematic summarizes the multifaceted properties of oncolytic viruses and their integration with modern immunotherapeutic strategies [151, 152, 153, 154]. The left panel demonstrates the selective replication of oncolytic viruses in malignant cells, sparing healthy and non‐immune cells, and inducing lysis and infection of neighboring tumor cells. The central panel illustrates the immune activation cascade at the tumor site after oncolytic viral replication, including immunogenic cell death (oncolysis), antigen and cytokine release, DC priming, CD8+ T cell activation, and migration to the tumor. Integration points between adoptive cell therapy and cancer vaccines are indicated: adoptive cell therapy enhances effector T‐cell infiltration and cytotoxicity. In contrast, cancer vaccines promote uptake and presentation of tumor antigens by DCs, amplifying T cell responses. The right panel depicts the use of transgene‐expressing oncolytic viruses to deliver therapeutic molecules within malignant cells. Together, this figure highlights how oncolytic virotherapy can synergize with additional immunotherapies to promote anti‐tumor immune responses and tumor. Panel (A) was generated by the authors based on information from the 3CA database [136] (https://www.weizmann.ac.il/sites/3CA/). Panels (B) and (C) were independently designed and generated by the authors using BioRender, based solely on information reported in the cited literature [143, 149, 150, 151, 152, 153, 154]. Panel (A) is generated using the ggplot2 package in R (version 4.3.2).

MSLN expression is also highly heterogeneous within individual tumors. For instance, the Human Protein Atlas dataset suggests that although a tumor may be positive for MSLN expression with strong staining, the staining may be limited to specific tumor cells, with percentages lower than 25% or between 25 and 75%. Expression levels higher than 75% occur rarely (Figures 4 and S9–S11). Thus, significant sections of the tumor might lack MSLN expression entirely. Its expression may be confined to the cellular cytosol or the nucleus. Notably, colon adenocarcinoma exhibited exclusive nuclear MSLN localization, whereas others showed mixed cytoplasmic and membranous retention. This intracellular compartmentalization poses a profound biophysical barrier to therapeutic efficacy [133, 134, 135]. To elucidate the molecular mechanism underlying this variability, we have also explored MSLN expression at single‐cell resolution across multiple cancer types using 3CA datasets [136]. MSLN expression is confined to neoplastic and epithelial cells, especially of ovarian and pancreatic cancer, and does not occur in stromal and immune cells (Figure 5A). This finding indicates that tumors are comprised of a heterogeneous population of MSLN‐positive and negative clones. Cellular compartmentalization, in turn, leads to heterogeneous MSLN expression, as observed by IHC, underscoring the importance of clonal heterogeneity in the development of MSLN heterogeneity. Earlier intratumor heterogeneity in ovarian cancer patients was also identified in 23% of the 168 primary tumors evaluated for MSLN expression. This percentage was lower in 154 cases of peritoneal carcinomatosis (12%) and even lower in 71 patients with lymph node metastasis (6%) [130]. In a single‐cell study, increased MSLN expression was observed exclusively in the GC_P02T tumor cell subcluster, consistent with its known upregulation in GC and other solid tumors [14, 120]. MSLN overexpression is reported to be mainly restricted to the tumor epithelium and is characterized by the absence of stromal fat tissue in the TME. At the clinical level, MSLN overexpression is closely related to TNBC subtype and N stage. Despite this relationship, MSLN overexpression does not significantly affect long‐term outcomes, including OS, disease‐specific survival, and disease‐free survival [86].

In summary, there is significant heterogeneity in MSLN expression among patients and even within individual tumors. Intrapatient heterogeneity suggests that a “one size fits all” approach will not work because only a subset of patients can benefit from the therapy. Furthermore, intratumoral heterogeneity, resulting from complex spatiotemporal compartmentalization, means that a therapeutic agent faces a constantly changing target, as elimination of one cell clone can lead to the dominance of another clone that lacks MSLN expression.

## 6 MSLN‐Targeted Therapy

Although heterogeneity has posed challenges in the clinic, the high cell‐surface expression of MSLN makes it an attractive therapeutic target. In recent years, numerous strategies, including immunotherapy, have been explored in preclinical models and early clinical trials [137] (Tables 2 and 3). Immunotherapy is a transformative cancer treatment that harnesses the immune system to eliminate tumors and maintain long‐term immune surveillance, thereby reducing the risk of relapse. Immunotherapy primarily regulates the immune microenvironment, enabling the immune system to effectively identify, target, and kill tumor cells through several key regulatory pathways [138, 139, 140]. Immunotherapy approaches can be broadly classified into passive and active modalities [141, 142, 143, 144, 145, 146, 147, 148] (Figure 5B). Passive immunotherapies deliver exogenous immune components that exert direct antitumor effects, eliminating the need for host immune priming. It may be nonspecific (such as adoptive cell transfer [ACT] of tumor‐infiltrating lymphocytes [TILs]) or specific (including engineered adoptive cell therapies, such as mAbs, CAR‐T, bispecific antibodies, and ADCs). In contrast, active immunotherapies stimulate the patient's own immune system, either nonspecifically, using immune checkpoint inhibitors, or via vaccines or oncolytic viruses that present tumor antigens to trigger targeted adaptive responses.

**TABLE 2:** Clinical landscape of MSLN‐targeted immunotherapy.

Approach	Mechanism	Clinical outcomes	Challenges	References
CAR T cells	Engineered to target surface MSLN; transient mRNA expression	Transient response in 1/10 refractory PDAC	Nonsustained responses; off‐target toxicity	NCT01355965 [74]
CAR T cells	Evaluation of feasibility, safety, and efficacy of anti‐MSLN CAR‐T cells	Treatment with mesothelin‐specific CAR‐T cells appears to be well tolerated and has shown promising results in inducing an antitumor effect in patients with PDAC.	Modest clinical efficacy Only two out of the six treated patients achieved “stable disease.”	NCT01897415 [128, 155]
CAR T cells	Anti‐MSLN CAR‐T cells (evaluated in MPM and PDAC, *n* = 2)	The cells may successfully induce an antitumor effect.	Regimen was entirely untolerated (steep trade‐off between efficacy and safety); resulted in one treatment‐related death, and the second patient experienced multiple Grade ≥3 adverse events.	NCT01355965 [128, 156]
CAR T cells	Anti‐MSLN CAR‐T + aldesleukin (Phase I/II for solid tumors, *n* = 15)	Poor efficacy: 0% objective response rate (93% progressive disease, 7% stable disease)	High toxicity (45% experienced serious adverse events); trial terminated early due to poor patient accrual.	NCT01583686 [128]
CAR T cells	huCART‐meso + VCN‐01 (Phase I for pancreatic and ovarian cancers, target *n* = 13)	N/A (trial ongoing; estimated completion 2038). Evaluating the feasibility of varied dosing schedules without lymphodepletion	N/A (data not yet available)	NCT05057715 [128]
CAR T cells	Intratumoral huCART‐meso cells (Phase I for TNBC, target *n* = 12)	N/A (trial ongoing; estimated completion 2038). Evaluating the feasibility and safety of intratumoral injection (without lymphodepletion)	N/A (data not yet available)	NCT05623488 [128]
CAR T cells	huCART‐meso (intravenous, intraperitoneal, or intrahepatic delivery) (Phase I for pancreatic cancer, target *n* = 18)	N/A (trial ongoing; estimated completion 2025). Evaluating the feasibility and safety of different administration routes without lymphodepletion	N/A (data not yet available)	NCT03323944 [128]
CAR T cells	huCART‐meso cells (intravenous and local delivery) (Phase I for mesothelioma, ovarian, and pancreatic cancers, *n* = 15 evaluated)	Median progression‐free survival was 2.1 months. The best overall response included 0% complete response, 0% partial response, 73% stable disease, and 27% progressive disease.	Safety concerns included seven Grade ≥3 adverse events in the studied cohort. Objective clinical responses not achieved (no complete response or partial response observed).	NCT03054298 [128, 157]
CAR T cells	huCART‐meso + anti‐CD19 CAR‐T cells (Phase I for PDAC, terminated, *n* = 3 evaluated)	Best overall response was 0% complete response, 0% partial response, 33% stable disease, and 67% progressive disease. Anti‐CD19 CAR‐T cells eradicated normal B‐cells.	Premature termination owing to lack of efficacy and poor funding. Administration of anti‐CD19 CAR‐T cells at the stated dosage does not improve the survival of the huCART‐meso cells. Objective responses, including complete and partial responses, have not been observed.	NCT02465983 [158]
CAR T cells	huCART‐meso cells (Phase I for PDAC, epithelial ovarian cancer, and MPM, *n* = 15 evaluated)	Progression‐free survival of 2.1 months was achieved. The best overall response consisted of 0% complete response, 0% partial response, 73% stable disease, and 27% progressive disease.	Dose‐limiting toxicity was observed in the cohort receiving 1–3 × 10^7^ cells/m^2^ without lymphodepletion. Did not achieve objective clinical responses (no complete response or partial response).	NCT02159716 [157]
CAR NK cells	Allogeneic CAR‐NKT cells simultaneously target ovarian cancer cells and the tumor microenvironment	Superior antitumor efficacy compared with CAR‐T cells, reduced cytokine release syndrome, no graft‐versus‐host disease, resistance to host immune rejection, and promising off‐the‐shelf potential	Potential on‐target, off‐tumor toxicity; optimization of CAR design and safety mechanisms; clinical validation required.	[159]
CAR NK cells	Allogeneic IL‐15‐enhanced CAR‐NKT cells mediate CAR‐ and NK receptor‐dependent cytotoxicity against pancreatic cancer cells	Superior tumor control, improved tumor homing, sustained effector function, reduced exhaustion, limited cytokine release syndrome, and absence of graft versus host disease (preclinical)	Clinical validation and optimization for clinical use	[160]
CAR NK cells	Engineered Allo15MCAR‐NKT cells by applying hematopoietic stem and progenitor cell technology together with feeder‐free differentiation technology in developing IL‐15‐enhanced mesothelin‐targeting CAR‐NKT cells	Allo15MCAR‐NKT cells showed excellent tumor control, effector function, minimal exhaustion, and a good safety profile, including the absence of graft‐versus‐host disease, in TNBC models.	Clinical validation and evaluation of long‐term persistence and memory potential are required.	[161]
CAR NK cells	Allogeneic CAR‐NKT cells target mesothelin‐positive UEC tumor cells and eliminate CD1d^+^ immunosuppressive cells, including tumor‐associated macrophages and myeloid‐derived suppressor cells.	Potent antitumor activity, effective TME modulation, minimal cytokine release syndrome, no graft‐versus‐host disease, and promising off‐the‐shelf therapeutic potential.	Clinical validation will be required for a more accurate estimation of the persistence, immune tolerance, and safety of allogeneic MCAR‐NKT cells.	[162]
IL‐7/CCL19 CAR‐T	CAR‐T cells engineered to secrete IL‐7 and CCL19 to enhance T cell proliferation and recruitment; targets GPC3 or MSLN	Phase I trial evaluating safety and efficacy in advanced hepatocellular, pancreatic, and ovarian carcinomas	Early‐phase study; efficacy and long‐term outcomes still under investigation	NCT03198546
				
TCR‐engineered T cells	Ex vivo TCR modification for MSLN recognition on MHC	Phase I safety in Stage IV PDAC	Terminated due to funding; no efficacy data	NCT04809766
Antibodies and ADCs	SS1P + pentostatin/cyclophosphamide; delays antibodies	3 partial responses (44–74%), 3 stable disease, 4 progressive disease	Grade 3 toxicities in 9% of patients; Grade 4 lymphopenias in all patients	NCT01362790
Antibodies and ADCs	LMB‐100 ± nab‐paclitaxel; MSLN‐targeting immunotoxin ± nab‐paclitaxel; Arm A: short infusion, Arm B: long infusion	Arm A: clinical activity; partial response in 1 patient with increased effector T cells and reduced Tregs; Arm B: no efficacy; both arms showed proliferation of CD4/CD8 T cells.	Arm A: severe capillary leak syndrome (CLS) linked to IFNγ and IL‐8 elevations; Arm B: reduced CLS but lack of efficacy.	NCT02810418
Antibodies and ADCs	Anetumab ravtansine (BAY94‐9343); MSLN‐targeted ADC conjugated to DM4; internalization induces microtubule disruption, cell‐cycle arrest, and apoptosis	Well tolerated; acceptable toxicity profile with every‐3‐week dosing	Not reported in Phase I; further efficacy data pending	NCT02485119
Antibodies and ADCs	DMOT4039A; Anti‐MSLN ADC conjugated to monomethyl auristatin E; disrupts microtubules to induce apoptosis	Six partial responses in PDAC/ovarian cancer	Dose‐limiting toxicities: Grade 3 hyperglycemia and hypophosphatemia	NCT01469793
Antibodies and ADCs	Anetumab ravtansine (AR) + bevacizumab (Bev) vs. paclitaxel + bevacizumab (Phase II, HGOC)	Control (paclitaxel + Bev): progression‐free survival: 12.7 months; partial response: 66%, stable disease: 20%. Experimental (AR + Bev): safe dose (recommended Phase 2 dose) established at 2.2 mg/kg. complete response: 4%, PR: 18%, stable disease: 50%.	Lower efficacy (AR + Bev): shorter PFS (5.3 vs. 12.7 mo) and increased disease progression (progressive disease: 28 vs. 14%) than control. Toxicity: high severe adverse events in both arms (29 Grade ≥3 treatment emergent adverse events in 28 AR patients vs. 26 in 29 control patients).	NCT03587311 [128, 163]
Antibodies and ADCs	AR + bevacizumab vs. paclitaxel + bevacizumab (Phase II, HGOC)	Control (paclitaxel + Bev): PFS: 12.7 months; PR: 66%, SD: 20%. Experimental (AR + Bev): Safe dose (recommended Phase 2 dose) established at 2.2 mg/kg. CR: 4%, PR: 18%, SD: 50%.	Lower efficacy (AR + Bev): shorter PFS (5.3 vs 12.7 mo) and increased disease progression (PD: 28 vs. 14%) than control. Toxicity: high severe adverse events in both arms (29 Grade ≥3 treatment emergent adverse events in 28 AR patients vs. 26 in 29 control patients).	NCT03587311 [128, 163]
Antibodies and ADCs	AR alone and in combination with gemcitabine (Phase II, solid tumors)	Efficacy (*n* = 9): overall survival: 34.1 months. Safety: 0% treatment‐related serious adverse events (TRSAEs)	Trial status: terminated (company strategy decision—only nine patients evaluated). Toxicity: 100% had TEAEs, 89% had treatment‐related AEs (treatment‐emergent adverse event), and 22% had treatment‐emergent serious AEs (TESAEs).	NCT03926143 [128]
Antibodies and ADCs	AR (Phase II, pancreatic cancer)	Efficacy (*n* = 14): time to progression: 2.1 months; stable disease: 14%. Posology: AR 6.5 mg/kg i.v. once every 3 weeks	Poor efficacy: 0% objective response (0% complete response, 0% partial response) and high rate of disease progression (PD: 86%). Toxicity (*n* = 18): 61% of patients experienced serious adverse events (SAEs).	NCT03023722 [128, 164]
Antibodies and ADCs	AR (Phase II, lung cancer)	Efficacy (*n* = 2): overall survival: 1.7 months; progression‐free survival: 1.3 months. Posology: AR i.v. once every 3 weeks	Trial terminated: ended early due to slow/insufficient accrual (only two patients evaluated). Poor efficacy: 0% objective response (0% complete response, 0% partial response). Toxicity (*n* = 2): 50% of patients experienced a serious adverse event (SAE) (respiratory failure).	NCT02839681 [128]
Vaccine	The assessment of the safety and efficacy of CRS‐207, along with pembrolizumab, ipilimumab, and tadalafil, shows that although CRS‐207 is included in the drug regimen, the study does not specifically focus on mesothelin (MSLN).	In PDAC, CRS‐207 was infused intravenously at a dose of 1 × 10^9^ colony‐forming units (CFU) on Day 2 of Cycles 1–6 (3‐week cycles). This regimen was combined with pembrolizumab infusion at a dose of 200 mg on Day 1 of Cycles 1 to 6, ipilimumab infusion at a dose of 50 mg on Day 1 of Cycles 1, 3, and 5, and tadalafil oral administration at 20 mg from Days 3 to 21 of Cycles 1 to 6. Safety and efficacy: not applicable		NCT05014776 [165]
Vaccine	Safety and efficacy of CRS‐207 with pembrolizumab	Gastric, gastroesophageal junction (GEJ), and esophageal, CRS‐207 (1 × 10^9^ CFU i.v.; Day 2 Cycle 1, Day 1 Cycles 2–4, then q6w) was given with pembrolizumab 200 mg i.v. (Day 1 each 3‐week cycle). In 5 patients, 80% experienced serious adverse events; median overall survival was 2.5 months, and median progression‐free survival was 1.2 months, with no observed responses (0% complete response/partial response/stable disease; 100% progressive disease in evaluable patients).	The study was stopped due to low enrollment and a lack of clinical activity observed in other CRS‐207 trials.	NCT03122548

Abbreviations: CAR, chimeric antigen receptor; PDAC, pancreatic adenocarcinoma; TNBC, triple‐negative breast cancer; VCN, oncolytic adenovirus.

**TABLE 3:** Preclinical strategies of MSLN‐targeted immunotherapy.

Approach	Mechanism	Clinical outcomes	Challenges	References
Adoptive cell transfer	Nonspecific lymphocyte expansion from tumors	Better PDAC outcomes with high T‐cell density	Suppressive TME (PD‐L1, Tregs); CD4+ dominance, mixed results	[166]
CAR T cells	HSV‐MSLN + MSLN‐CAR T cells (murine PDAC); Oncolytic HSV1 engineered to express MSLN, combined with MSLN‐CAR T cells; virus delivers MSLN to tumor cells and activates CAR T cells.	In vitro: efficient MSLN delivery and CAR T activation; in vivo: tumor growth suppression, improved survival, increased unexhausted CD8^+^ T cells, reduced Tregs, enhanced CAR T activity, dendritic cell activation, and priming of naïve CD8^+^ T cells in tumor‐draining lymph nodes	Preclinical study; translational potential for solid tumor immunotherapy	[167]
CAR T cells	CAR designs employing an scFv derived from SS1P that recognizes MSLN, with a 41BB costimulatory domain and a CD3ζ signaling domain‐stem/progenitor cells.	The CARs designed in this study recognize MSLN‐expressing AML blasts and leukemic stem‐cell‐enriched CD34^+^CD38^−^ cell fractions, but not normal hematopoietic cells.		[168]
Antibodies and ADCs	BMS‐986148 ± nivolumab; MSLN‐targeted ADC delivering a cytotoxic payload; enhanced efficacy with anti‐PD‐1 antibody nivolumab in preclinical studies	Durable responses in solid tumors of some patients	Limited PDAC‐specific data	NCT02341625, NCT02884726
Antibodies and ADCs	Bispecific (LicMAb); SIRPα/CD47 blockade with MSLN targeting	Enhances phagocytosis in PDAC models	Preclinical; off‐tumor minimization	[169]

Abbreviations: ADC, antibody–drug conjugates; CAR, chimeric antigen receptor; PDAC, pancreatic adenocarcinoma; TNBC, triple‐negative breast cancer.

### 6.1 Passive Immunotherapy Strategies

#### 6.1.1 Chimeric Antigen Receptor T‐Cell Therapy

In ACT immunotherapy, lymphocytes—either naturally occurring or genetically engineered—are infused into patients to target tumors. Acting as a “living drug,” these cells proliferate upon encountering their specific antigen, enabling recognition and destruction of cancer cells [170]. The TILs act mainly in a nonspecific manner; hence, they are unable to target any particular antigen, such as MSLN [147, 148]. However, this is not the case with CAR‐T and T‐cell receptor (TCR)‐modified T cells, which can specifically target antigens [171]. TCR‐engineered T cells are produced by ex vivo modification of patient T cells to express receptors that recognize tumor antigens presented by MHC Class I or II molecules [172]. A Phase I clinical trial (NCT04809766) evaluated the safety and efficacy of autologous MSLN‐specific TCR T cells in patients with Stage IV PDAC. Following leukapheresis and lymphodepletion with bendamustine, fludarabine, and cyclophosphamide, participants received three infusions of TCR‐T_MSLN_ cells at 21‐day intervals. Primary endpoints focused on safety and dose‐limiting toxicities. In contrast, secondary endpoints included “OS”, “objective response rate” (ORR), and “PFS”, with a target ORR of 20% among 15 participants. The trial, however, was terminated prematurely due to a lack of funding [173].

CAR‐T cells are engineered to recognize the cell surface antigen MSLN, which is associated with tumor invasiveness [174, 175]. Previously, Jiang et al. reported that anti‐MSLN CAR‐T cells, MSLN‐28BBZ, and MSLN‐28Z, effectively suppressed tumor growth and infiltrated MSLN‐positive patient‐derived xenograft models of PDAC [176]. CAR‐T cells targeting MSLN are under clinical evaluation for PDAC, with early reports showing promising antitumor immune responses [74, 156]. In a Phase I trial (NCT01355965), autologous T cells were transiently engineered via in vitro transcribed mRNA to express an MSLN‐specific CAR containing CD3ζ and 4‐1BB costimulatory domains. Patients with chemotherapy‐refractory metastatic PDAC received three infusions per week for 3 weeks. Clinical activity was limited: one of ten patients showed a complete metabolic response in the liver by FDG‐PET/CT after three CAR‐T doses, but this response was not durable, and the patient ultimately succumbed to disease progression [155]. Furthermore, next‐generation MSLN‐CAR designs, such as potent anti‐MSLN hYP218 CAR‐T cells, have demonstrated enhanced tumor infiltration and persistence, thereby improving their antitumor efficacy both in vitro and in vivo [177]. CAR‐T cell therapies targeting MSLN, alone or in combination with CD19, have shown safety and tolerability in patients with metastatic PDAC [158]. Additionally, in orthotopic animal models, MSLN‐specific CAR‐T cells have been shown to control tumor growth effectively [178]. In mice with highly aggressive PDAC, combining MSLN‐redirected CAR‐T cells with TNF‐α/IL‐2‐armed oncolytic adenoviruses lead to significant tumor regression [179]. In a Phase I clinical trial, T cells engineered to express MSLN‐specific CARs were administered to six patients with chemotherapy‐refractory metastatic PDAC. The therapy was well tolerated, with no severe toxicities, and resulted in stable disease in two patients. Additionally, one patient exhibited a marked reduction in tumor metabolic activity, underscoring the potential antitumor efficacy of these engineered T cells [180].

Preclinical studies have also emphasized the promise of CAR‐NK cell‐based therapies in PDAC. For example, CAR‐NK cells targeting ROBO1, when combined with radiation therapy, exhibited enhanced antitumor efficacy in an orthotopic mouse model of human PDAC [181]. Similarly, a novel NK cell‐based therapy targeting prostate stem cell antigen (PSCA) effectively suppressed PSCA^+^ PDAC in both in vitro and in vivo models, without causing systemic toxicity [182]. Moreover, in a separate mouse model, CAR‐NK cells targeting MSLN, when combined with the STING agonist cGAMP, produced significant inhibition of tumor growth and extended survival [183]. Building on these preclinical results, two clinical trials are currently investigating ROBO1‐ and MUC1‐specific CAR‐NK cell therapies in patients with PDAC (NCT03941457, NCT02839954), with primary endpoints assessing safety and ORR. In another study, the investigators showed that MSLN cell‐surface expression was restricted to AML blasts and absent from normal hematopoietic lineages in individual patients [168]. NK‐92 cell‐based therapies currently being evaluated in clinical trials have shown promising efficacy and safety profiles in treating various cancers, including AML. In addition, the investigators revealed that CAR‐NK cells targeting MSLN were effective at killing MSLN‐expressing AML blasts in vitro and ex vivo in animal models of AML.

Recent literature has revealed the benefits of using allogenic MSLN‐specific CAR‐NKT (AlloMCAR‐NKT) cells in various solid tumors. It has been shown that, in ovarian cancer, compared with traditional CAR‐T cells, AlloMCAR‐NKT cells have exhibited superior effects, better tumor homing ability, and safety without cytokine release syndrome or signs of graft‐versus‐host disease or host rejection [159]. The Allo15MCAR‐NKT cells have also demonstrated strong anticancer activity against pancreatic cancer through both CAR‐dependent and NK receptor‐based cytotoxic mechanisms, thereby facilitating increased infiltration into tumors, prolonged effector function, decreased exhaustion, and improved tumor control in experimental models. Moreover, the Allo15MCAR‐NKT cells displayed an advantageous safety profile, characterized by limited occurrence of cytokine release syndrome and no graft‐versus‐host disease [160]. In yet another study, investigators engineered Allo15MCAR‐NKT cells by applying hematopoietic stem and progenitor cell technology together with feeder‐free differentiation technology in developing IL‐15‐enhanced MSLN‐targeting CAR‐NKT cells. This resulted in the production of cells with strong antitumor activity against TNBC cells via both CAR‐mediated and natural killer receptor‐mediated cytotoxicity and selective elimination of CD1d^+^ immunosuppressive cells in the TME. The preclinical data on Allo15MCAR‐NKT cells showed excellent tumor control, effector function, minimal exhaustion, and a good safety profile, including the absence of graft‐versus‐host disease, in TNBC models [161]. In yet another research study, scientists created allogeneic MSLN‐directed CAR‐NKT cells via engineering of hematopoietic stem and progenitor cells and ex vivo differentiation culture. The cells have shown excellent ability to target not only uterine endometrioid carcinoma tumor cells but also CD1d^+^ immunosuppressive cells of the TME, which included tumor‐associated macrophages (TAMs) and myeloid‐derived suppressor cells. Compared with the classical CAR‐T cells, AlloCAR‐NKT cells showed an outstanding safety profile with minimal cytokine release syndrome and absence of graft‐versus‐host disease [162]. This work suggests that it may be worthwhile to evaluate the clinical effectiveness of MSLN‐targeting CAR NK cells in patients with relapsed/refractory AML.

For CAR‐T cells to fight cancerous cells, they must come into contact with antigens on the surface of tumor cells. To improve CAR‐T cell trafficking and infiltration into solid tumors, CAR‐T cells have been genetically modified to overexpress cytokine and chemokine receptors [184, 185, 186]. Early studies have also demonstrated that several proinflammatory cytokines have potent antitumor effects in animal models [187]. This led to the clinical use of recombinant interferon‐alpha and interleukin‐2 to treat various malignancies, although their efficacy was modest. Jin et al. found that tumors exhibit high expression of IL‐8, CXCL1, and CXCL2. They modified CAR‐T cells to express CXCR1 and CXCR2, receptors for IL‐8. These modifications led to improved CAR‐T cell migration toward tumors and increased antitumor activity against solid tumors [188]. Hodgkin lymphoma involves Reed‐Sternberg cells that secrete CCL17 and CCL22, which bind CCR4 on T helper cells and regulatory T cells, thereby driving their migration to the tumor site [189]. Savoldo et al. found that CCR4.CD30 CAR‐T cells, which express both CCR4 and CD30 on their surface, have better homing properties and improved antilymphoma effects compared with CD30 CAR‐T cells without CCR4 expression [190]. In another clinical trial (NCT03602157), CCR4.CD30 CAR‐T cells were found to be effective in treating relapsed/refractory classical Hodgkin lymphoma, with greater migration to the skin resulting in enhanced activity against CD30^+^ cutaneous T‐cell lymphomas [190]. Similarly, engineering CAR‐T cells to express the CCL2 receptor, CCR2b, increased tumor infiltration, resulting in a 10‐fold increase in anti‐GD2 CAR‐T cell infiltration in neuroblastoma xenografts and a 12‐fold increase in MESO models compared with unmodified CAR‐T cells [191, 192]. Li et al. further showed that CAR‐T cells coexpressing IL‐7 and CCR2b exhibited improved survival and enhanced infiltration in glioblastoma and melanoma models [193]. In addition, CCR2b‐modified anti‐MSLN CAR‐T cells exhibited enhanced migration and tumor infiltration, with superior antitumor activity, particularly in non‐small‐cell lung carcinoma [194]. Recently, in preclinical studies, efforts have been made to use novel cytokine/chemokine receptor approaches to stimulate CAR‐T cell infiltration into tumors. Specifically, CAR‐T cells targeting CLDN18.2 antigen that were genetically modified to coexpress IL‐7 and CCL21 (referred to as “7×21” CAR‐T cells) showed enhanced proliferation and chemotaxis, resulting in improved anticancer effects for different cancers, such as PANC02 (pancreatic cancer), E0771‐A2 (breast cancer), and Hepa1‐6‐A2 (liver cancer) [195].

Cytokine‐based therapies are limited by poor targeting and an incomplete understanding of cytokine effects across cancer types. Antibody–cytokine fusions have been used preclinically to direct cytokines to the TME, but their large size reduces tissue penetration and prolongs circulation. To overcome this, a group of researchers developed alpaca‐derived heavy chain‐only antibody fragments (VHHs) targeting PD‐L1 [196]. In cancer patients, PD‐L1 is primarily expressed in the TME, on both tumor cells and infiltrating myeloid cells. At ∼15 kDa, VHHs penetrate tumors more effectively than full‐sized antibodies. Previous attempts to deliver IL‐2 via antibody–cytokine fusions were limited because IL‐2's high receptor affinity and its widespread expression on receptors in the blood, spleen, and liver led to distribution dominated by IL‐2 rather than by the antibody's targeting. In a Phase I trial (NCT03198546), the safety and efficacy of CAR‐T cells engineered to secrete IL‐7 and CCL19 (7×19) were evaluated in patients with advanced hepatocellular, pancreatic, or ovarian carcinomas expressing GPC3 or MSLN. Remarkably, a hepatocellular carcinoma patient achieved complete tumor remission 30 days after intratumoral injection of anti‐GPC3‐7×19 CAR‐T cells, and a patient with PDAC exhibited near‐complete remission 240 days following intravenous infusion of anti‐MSLN‐7×19 CAR‐T cells. These findings indicate that coexpression of IL‐7 and CCL19 substantially enhances CAR‐T cell activity against solid tumors, highlighting a promising strategy for PDAC and other malignancies.

Additionally, oncolytic herpes virus type 1 (oHSV1) can be genetically engineered to express specific surface proteins on tumor cells, further enhancing its therapeutic versatility. In one study [167], an HSV‐MSLN virus derived from a clinical HSV‐1 strain was combined with MSLN‐CAR‐T cells to treat murine PDAC. HSV‐MSLN efficiently delivered MSLN to tumor cells and activated CAR‐T cells in vitro. In vivo, the combination therapy suppressed tumor growth, improved survival, and altered the TME by increasing the number of unexhausted CD8^+^ T cells, reducing Treg numbers, and enhancing CAR‐T cell activity. It also promoted dendritic cell activation and the priming of naïve CD8+ T cells in tumor‐draining lymph nodes, highlighting the potential of oncolytic virotherapy combined with CAR‐T cells to treat solid tumors.

#### 6.1.2 Chimeric mAbs and ADCs

mAbs are laboratory‐engineered proteins designed to precisely recognize and bind specific antigens, mimicking the immune system's natural ability to target pathogens and cancer cells [197]. Their versatility increases when combined with cytotoxic drugs, such as ADCs, enabling the selective killing of cancer cells while sparing healthy tissue [198]. Another promising strategy involves immunotoxins, which fuse bacterial or plant‐derived toxins with targeting modules to achieve potent anticancer effects [199]. Most immunotoxins under preclinical or clinical investigation are constructed as fusion proteins linking a toxin to an antibody fragment. In contrast, those based on full‐length antibodies remain less explored, despite the Fc domain's critical role in maintaining IgG serum levels and mediating immune responses.

SS1P is the first immunotoxin targeting MSLN and shows potent cytotoxicity against MESO and ovarian cancer cell lines [137]. PDAC models, however, were resistant primarily to SS1P monotherapy, prompting exploration of combination strategies. For example, SS1P combined with gemcitabine lacked synergy in vitro on A431/K5 cells but resulted in complete tumor regressions in A431/K5 xenografts [200]. Similarly, Matoo et al. demonstrated that the PDAC cell line KLM‐1 was resistant to SS1P alone; however, the addition of the PKC inhibitor enzastaurin enhanced immunotoxin activity 10‐fold in a dose‐dependent manner [201]. Resistance was also overcome by combining SS1P with the BH3‐mimetic ABT‐737, significantly increasing cell death in KLM‐1, BxPc‐3, and Panc 3.014 cells [202]. However, a key limitation of SS1P in PDAC is the host immune system's generation of neutralizing antibodies after repeated dosing [15].

To address this, immunosuppressive regimens such as pentostatin and cyclophosphamide, which target T‐ and B‐lymphocytes, have been used to prevent antibody formation, resulting in tumor regression in MESO models [203, 204]. Clinical studies further explored these strategies [11]. For instance, in the Phase II trial NCT01362790, 10 patients with chemotherapy‐refractory MESO received SS1P in combination with pentostatin and cyclophosphamide. Among them, three patients achieved partial responses (30–74%), three had stable disease, and four experienced disease progression. Grade 3 toxicities, including noncardiac chest pain, pleuritic pain, and back pain, occurred in 9% of patients, whereas Grade 4 lymphopenias related to pentostatin or cyclophosphamide were observed in all participants. The immunosuppressive regimen delayed the development of neutralizing antibodies, enabling extended SS1P therapy. Separately, SS1P combined with pemetrexed and cisplatin was tested in 20 chemotherapy‐naive patients with advanced MPM (NCT01445392), yielding 12 partial responses, three stable disease, and five progressive diseases. Biomarker changes in MSLN, MPF, and CA125 correlated with response, decreasing in patients with partial responses and increasing in those with progressive disease. LMB‐100 is another MSLN‐targeting immunotoxin evaluated in a Phase 1/2 trial (NCT02810418) with or without nab‐paclitaxel [205]. In Arm A (*n* = 20), short infusions with nab‐paclitaxel showed clinical activity. Still, they were limited by severe capillary leak syndrome (CLS), which is characterized by rapid elevations in IFNγ and IL‐8 and the expansion of activated CD4/CD8 T cells. The only partial responder also showed increased effector T cells and reduced Tregs. In Arm B (*n* = 20), long infusions (24–48 h) reduced CLS but failed to demonstrate efficacy, even with nab‐paclitaxel. Both arms demonstrated treatment‐driven proliferation of CD4 and CD8 T‐cell subsets, indicating that LMB‐100 induces systemic immune activation, whereas CLS remains the primary barrier to therapeutic success.

Anetumab ravtansine (BAY94‐9343) is a MSLN‐targeted ADC conjugated to the tubulin inhibitor DM4 [206]. Once internalized by MSLN‐positive tumor cells, DM4 induces cell‐cycle arrest and apoptosis. In a Phase I trial (NCT02485119), BAY94‐9343 was well tolerated with an acceptable toxicity profile when administered every 3 weeks. A separate Phase I study (NCT01439152) reported partial responses in 19% of patients and stable disease in 47%. BAY94‐9343 is currently being evaluated in multiple Phase I/II trials, including Phase II studies in pancreatic (NCT03023722) and lung cancers (NCT02839681). Another ADC, DMOT4039A, an anti‐MSLN antibody linked to monomethyl auristatin E, was tested in patients with PDAC and ovarian cancer (NCT01469793), where dose‐limiting toxicities included Grade 3 hyperglycemia and hypophosphatemia. Six patients achieved partial responses at doses of 2.4–2.8 mg/kg administered every 3 weeks. Additional MSLN‐directed ADCs, such as BMS‐986148 and MDX‐1204, remain under investigation. These results support continued development of MSLN‐directed therapies for advanced cancers.

### 6.2 Active Immunotherapy Strategies

Immune checkpoint blockade (ICB) is a promising strategy to enhance antitumor responses. However, monotherapy trials have largely failed, likely due to low immunogenicity and a “cold” TME [207, 208, 209]. Tumors exhibit heavy myeloid cell infiltration, a paucity of CD8^+^ effector T cells, and low expression of granzyme B and IFN‐γ. These features reflect impaired adaptive T‐cell immunity and poor responsiveness to checkpoint inhibitors. Despite this, studies have provided valuable insights for future therapies. For instance, ipilimumab, a CTLA‐4 inhibitor, is the first United States Food and Drug Administration (US FDA)‐approved checkpoint inhibitor and has been shown to improve OS in metastatic melanoma [210]. This was followed by the approval of the PD‐1 inhibitor nivolumab for advanced melanoma, which demonstrated durable tumor remission, favorable OS, and acceptable long‐term safety.

Therapeutic cancer vaccines are designed to generate tumor‐specific CD4^+^ and CD8^+^ T cells capable of mounting strong and durable antitumor responses. These vaccines include whole‐tumor‐cell vaccines, DC vaccines, peptide vaccines, DNA vaccines, and mRNA vaccines [175, 211]. Tumor cell vaccination is a straightforward immunotherapy approach providing both CD4+ and CTL epitopes. However, cancer vaccines differ from conventional immunotherapies in that they target both surface and intracellular tumor antigens [175, 211]. Their main goal is to induce stem‐like memory T cells that provide durable immune memory. These vaccines deliver tumor antigens via cell‐based platforms, peptides, or nucleic acids, often in conjunction with immune adjuvants that activate dendritic cells. Once activated, DCs present antigens on MHC class I and II molecules to naïve T cells, while costimulatory signals, such as CD28 on T cells interacting with CD80/CD86 on DCs, drive T cell activation, proliferation, and differentiation. Once primed, effector T cells migrate to tumors to recognize and eliminate cancer cells. To date, only one therapeutic cancer vaccine, sipuleucel‐T (PROVENGE), has received US FDA approval, providing a modest survival benefit of approximately 4 months in patients with prostate cancer.

There have been two clinical trials on vaccines based on bacterial vectors [212]. CRS‐207 is a live attenuated *Listeria monocytogenes* strain genetically modified for expression of MSLN [213]. JNJ‐64,041,757 (ADU‐214) is a live attenuated, double‐deleted Listeria monocytogenes strain expressing MSLN. CRS‐207 was evaluated in patients suffering from PDAC, MESO, NSCLC, and ovarian cancer, with tolerable safety and immunological activity. The drug JNJ‐64,041,757 was studied both as monotherapy and in combination with nivolumab in two trials (NCT03371381 and NCT02592967), and both have been discontinued due to a lack of clinical benefit. However, despite the disappointing results of the above trials, research into neoantigen DNA vaccines continues. There is also ongoing research into alternative preclinical vaccines, such as Meso‐VAX, along with AAV‐IL‐12 and CTGF/MSLN DNA vaccines [214, 215].

Oncolytic viruses are cancer therapies that specifically infect and replicate in tumor cells while sparing normal cells (Figure 5C). They target cancer through cellular receptors, tumor‐suppressor defects, or engineered viral modifications. Oncolytic viruses ‐ induce cell death, often in an immunogenic manner, thereby releasing tumor antigens and stimulating antitumor immune responses [216]. Their design must carefully balance viral activity, the TME, and host immune defenses. To date, two genetically engineered “oncolytic herpes simplex virus type 1” (oHSV1) therapies have been approved: “talimogene laherparepvec” (T‐VEC) for melanoma by the US FDA in 2015, and G47Δ for glioblastoma in Japan in 2022 [167]. Both clinical and preclinical studies suggest that oHSV1 holds therapeutic potential for treating PDAC [217, 218]. These studies demonstrate that oHSV1 can soften the dense PDAC tumor stroma and convert the immunologically “cold” TME into a “hot” one [167].

Another oncolytic adenovirus, namely, VCN‐01, is engineered to replicate in cancer cells with a defective RB1 pathway [175, 219]. It produces hyaluronidase, which enhances viral spread within tumors and promotes the infiltration of chemotherapy drugs and immune cells. In animal models of PDAC, VCN‐01, when combined with chemotherapy, showed improved anticancer effects. Its hyaluronidase effectively disrupts the tumor stroma, facilitating delivery of therapies including chemotherapeutics and antibodies. A clinical study demonstrated that VCN‐01 can be safely administered intravenously in PDAC patients, with predictable and manageable adverse events. Intravenous VCN‐01 showed a favorable tolerability profile [220], providing a solid foundation for future use of oncolytic virotherapy in PDAC immunotherapy. Several clinical trials are also investigating oncolytic virus‐based therapies in PDAC. For instance, a Phase I/II study showed that intratumoral LOAd703, an adenovirus expressing TMZ‐CD40L and 4‐1BB ligand, combined with nab‐paclitaxel/gemcitabine was safe and feasible in unresectable or metastatic PDAC, achieving a 44% ORR and 94% disease control rate at the highest dose (NCT02705196). Additionally, pelareorep, an oncolytic virus, plus pembrolizumab demonstrated modest efficacy in unselected patients, with a 42% clinical benefit rate, and notable immunological changes in responders, including decreased VDAC1 expression in peripheral CD8+ T cells and reduced peripheral CD4+ Treg levels (NCT03723915).

In brief, the clinical evolution of MSLN‐targeted agents, including CAR‐T cells, ADCs, cancer vaccines, and oncolytic viruses, exemplifies the advantages and drawbacks of contemporary immunotherapy strategies, thereby confirming MSLN as an acceptable and druggable target. However, clinical efficacy can be limited by the complex nature of cancers, including heterogeneous MSLN expression, MSLN shedding as a soluble decoy receptor, and an immunosuppressive TME [207, 221, 222]. Additionally, antigen heterogeneity [223, 224, 225, 226, 227] and tumor immune evasion [228, 229, 230] are contributing factors for drug resistance and poor clinical durability. In this regard, future efforts should focus on developing novel strategies to overcome the obstacles posed by tumor heterogeneity, the stroma, and immune suppression.

## 7 Translational Challenges and Future Directions

As evidenced by the disparate results of currently available MSLN‐targeting modalities, fully harnessing their benefits requires overcoming significant biological hurdles. The first and most crucial obstacle, in many cases, is represented by the immunosuppressive properties of the TME, with high stromal content, more myeloid and regulatory T cells, and lower numbers of CD8+ T cells resulting in a “cold” immune environment, limiting antitumor actions [207, 221, 222]. Other barriers include antigen heterogeneity [223, 224, 225, 226, 227] and tumor immune escape [228, 229, 230] strategies, thereby reducing their effectiveness. There is ample evidence to show that the TME plays an important role in the development, progression, and treatment resistance of almost all tumors [13]. While the TME varies in composition and characteristics depending on the type of cancer, some common features are shared across cancers. The TME consists mainly of cancer‐associated fibroblasts (CAFs) and TAMs that interact extensively with cancer cells and other TME components. These interactions often promote cancer growth and survival by secreting immune‐suppressing factors and activating survival mechanisms. Several studies have shown that MSLN expression is correlated with specific characteristics of the TME. For instance, Malla et al. showed that high MSLN expression in CRC is positively correlated with low IFN‐γ expression, high HAVCR2 and PD‐L1 expression, and increased Treg cell infiltration in the TME [231].

MSLN expression in PDAC was associated with decreased CD8+ T cell infiltration, a lower lymphocyte infiltration score, and reduced cytolytic function [232]. Previous studies have indicated that MSLN is shed from tumors and is present at higher levels in serum from MESO and ovarian cancer patients. Antigen shedding is an established property of cancer cells that plays an important role in defining their interactions with therapies. Other studies have also found that MSLN shedding inside tumors may act as a hindrance to successful treatment with SS1P. Significantly, changes in MSLN levels in tumors following chemotherapy were accompanied by a lower antigen load, leading to increased SS1P penetration [233]. Moreover, it has been documented that soluble MSLN tends to accumulate in the microenvironment of PDAC cells, potentially acting as a decoy for MSLN‐targeted drugs and thereby reducing their effectiveness. This theory is derived from the finding that individuals with PDAC who have elevated MSLN levels in tumor biopsy samples have unusually low MSLN levels in the bloodstream, suggesting that MSLN shed by these cells is trapped in the TME. Despite numerous studies validating this theory, it is unclear how soluble MSLN is maintained in the TME [234]. Patients with high MSLN mRNA expression in resected tissue samples showed chemoresistance to platinum and cyclophosphamide therapy compared with chemosensitive patients with lower MSLN expression [28, 212]. Moreover, MSLN has been found to bind strongly to mucin 16 (CA125) on the cell membrane [234]. This process might enable ovarian cancer cells to adhere to mesothelial cells in the peritoneal cavity, thereby promoting peritoneal metastasis [109]. Additionally, increased MSLN expression in pancreatic carcinoma cell lines is correlated with several mechanisms of apoptosis resistance, such as TNF‐α‐induced apoptosis [32] and anoikis‐induced apoptosis [37], as well as decreased sensitivity to paclitaxel treatment [235]. However, many questions about MSLN immunosuppressive function remain open. In particular, the role of MSLN in modulating other immune mediators, including neutrophils, NK cells, and APCs, remains poorly understood [8].

In some cases, tumor recurrence may be attributed to heterogeneity in tumor antigen presentation [223, 224, 225]. To prevent antigen heterogeneity and avoid antigen escape, one approach is to design a CAR that recognizes multiple antigens by employing two single‐chain variable fragments (scFvs) in the antigen‐recognition segment. Tandem CARs have been tested in hematological cancers and solid tumors and, in some cases, have shown better tumor control than coadministration of monospecific CAR‐T cells [234, 236, 237]. In one study [223], scientists constructed the tandem CAR vector, TanCAR1, capable of simultaneously binding MSLN and the MUC16 ectodomain (MUC16ecto) without steric hindrance. The authors showed that the use of TanCAR1 provided better control of heterogeneous tumor models, both in vitro (including two‐ and three‐dimensional systems) and in vivo, than monospecific CAR‐T therapy could. From a mechanistic perspective, the experiment demonstrated that using tumor cells expressing varying amounts of both antigens led tandem CAR‐T cells to preferentially bind to one antigen at a time; however, tumor lysis depended on antigen density. It means that tumor killing depends on the antigen density on tumor cells. Overall, the results of the current experiment suggest that developing tandem CAR therapy may require some trial and error to design an efficient construct for each case, as the main determinants of efficacy may be antigen heterogeneity and density. Traditional CAR‐T cells usually target a single tumor antigen and are thus prone to variability in its presentation at different spatial and temporal locations [227]. To overcome this problem, an adapter CAR‐T (AdCAR‐T) approach was developed. This involves conjugating biotin to one end of the adapter molecule that binds CAR, while the other end targets multiple antigens on AML cells. The adaptability of this system enables targeted variation in the number and types of adapter molecules, resulting in greater specificity than that of standard single‐antigen CAR‐T cells. It has been shown that AdCAR‐T cells can target multiple antigens presented by AML cells, thereby avoiding immune escape due to antigen heterogeneity [238, 239].

The discovery of immune evasion mechanisms has also been highly instrumental in informing modern immunotherapy. One such important mediator of immunosuppression, which is becoming more apparent today, is MSLN. It is known to be a central driver of the immunosuppressive TME, for instance, through CD24‐mediated activation of M2 macrophages and the exclusion of CD8+ cytotoxic T cells [240]. Immune responses against MSLN have also been observed in individuals with BM, suggesting that host immunity can contribute significantly to managing tumor growth [241]. T cell responses specific to MSLN epitopes can therefore be harnessed through immunotherapy, particularly in patients devoid of natural anti‐MSLN responses. One study highlights the role played by MSLN in helping high‐grade serous ovarian cancer (HGSOC) cells evade the host immune system [138]. MSLN protein levels were significantly increased in HGSOC and were associated with poor clinical outcomes and reduced sensitivity to ICB treatment. Functionally, MSLN‐mediated activation of the Wnt/β‐catenin pathway increases CD24 levels and the protumorigenic phenotype of TAMs, thereby impairing T lymphocyte function within the TME. It was found that a feedback circuit exists in which MSLN upregulates Wnt/β‐catenin activity. Interestingly, knockdown of MSLN protein reduced CD24 expression and restored cytotoxic T cell functionality, improving patient sensitivity to PD‐1 blockade in mouse models. In summary, MSLN plays an important role in immune evasion in ovarian cancer, and inhibition of MSLN, in combination with ICB, can serve as an effective treatment modality for HGSOC.

Another major challenge is a high risk of off‐tumor toxicities [140, 242, 243, 244, 245]. To achieve optimal tumor destruction, CARs use high‐affinity scFvs as their targeting domains. However, doing so increases the risk of off‐tumor cytotoxicity because CAR T cells are more sensitive than mAbs in recognizing antigens on normal tissue surfaces [246]. For instance, a Phase I study [247] involving a highly active, fully human anti‐MSLN CAR T‐cell approach led to an observation that high doses given intravenously were accompanied by severe adverse effects, including pulmonary toxicity leading to death in one patient. It was found that the observed effects were off‐target but on‐tumor, caused by CAR T‐cell activity against nonmalignant lung tissue expressing low levels of MSLN. Given the potential for MSLN expression to be dynamically regulated during inflammation and fibrosis, subsequent MSLN trials should account for these factors. Another dose‐escalation trial using M5 CAR T cells proved ineffective across a wide range of solid cancers [248]. Although these cells successfully engrafted and homed in almost all patients (nine out of 14), they exhibited poor survival and were associated with only transient stable disease, without achieving any objective response. Moreover, pulmonary toxicity was observed at the dose level of 3 × 10^8^ cells/m^2^. This suggests that subsequent improvements would require loco‐regional administration or redesigning of these CAR T cells. Similarly, a study investigating the efficacy of the MSLN‐specific single‐domain TCR fusion protein (MH1) showed variable responses and dose‐dependent toxicity [249]. Safety concerns such as these underscore the need to develop safer MSLN‐targeted CAR T cells and more reliable preclinical models before proceeding to human trials. To address these toxicology concerns, affinity‐tuned MSLN nanobody CAR T cells were developed [250]. High‐affinity CAR T cells in mouse models were unable to migrate into tumor cells and caused fatal on‐target/off‐tumor toxicity. On the other hand, a reduction in binding affinity to micromolar levels enabled efficient migration of CAR T cells into the TME and increased specificity for MSLN‐high cells. Together, these factors significantly limit the effectiveness of therapies targeted against MSLN. Therefore, finding ways to enhance therapeutic efficiency in cancer treatment by increasing specificity for tumor tissue is an urgent scientific task.

Further efforts are underway to explore new delivery technologies and logic‐gated engineering to minimize severe toxicities [8]. Nanoencapsulation of drugs allows for controlled release and targeting, leading to a reduction in their systemic levels [251]. In addition, genetic engineering of CAR T cells to enable conditional activity allows dynamic control of CAR expression in response to tumor microenvironmental cues. IF‐THEN logic‐gated CAR T cells are programmed to be inactive until exposed to CAFs, which trigger the expression of MSLN‐CAR [252]. Additionally, the intracellular signaling cascades and epigenetic mechanisms involved in immune modulation require further study. In addition to understanding its immunomodulatory mechanism, another major hurdle to translating MSLN into clinical use is its strong context dependence across different cancers [8]. While its general role in oncogenesis is relatively well established, the prognostic significance and biological functions of MSLN vary widely across cancers.

Nevertheless, one of the main challenges in translating these advances into clinical practice is the high degree of contextual dependence of MSLN across different cancers. While the general role of MSLN in oncogenic processes is reasonably well known, its prognostic value and biological functions vary considerably across cancer types; for instance, it promotes tumor growth in PDAC [73] and helps tumors evade the immune system in HGSOC [138]. To account for such heterogeneity, patient stratification based on biomarker testing would be necessary: standard MSLN testing procedures, such as IHC criteria and liquid biopsy cut‐off points, need to be developed to ensure consistent stratification across different cancers. Coupling MSLN testing with other biomarkers, such as PD‐L1 expression [127], and tumor mutational burden [253], may be beneficial for selecting patients for MSLN‐directed therapy, especially when combined with immunotherapies like checkpoint inhibitors. Eventually, the future success of MSLN‐related clinical research will depend on how effectively we overcome these translational hurdles.

## 8 Conclusion and Prospects

In conclusion, MSLN is a well‐established biomarker and immunotherapy target in several cancers, including those of the pancreas and ovary, as well as MESO. Studies have shown that the MSLN protein promotes cancer progression through mechanisms that influence cell proliferation, metastasis, immune regulation, and resistance to apoptosis. Overexpression of MSLN in cancer patients, along with its scarcity in healthy individuals, has led to the development of numerous anti‐MSLN therapies, including CAR‐T cells, ADCs, and antibodies. However, a key interpretation emerging from clinical and preclinical studies is that MSLN expression alone is insufficient to predict therapeutic success. Despite strong rationale and encouraging early‐phase responses, clinical efficacy remains heterogeneous and often transient. This pattern suggests that MSLN should not be viewed as a static surface antigen, but rather as part of a dynamic tumor ecosystem influenced by both intrinsic tumor biology and microenvironmental constraints.

Regarding translational challenges, the studies suggest that future MSLN‐based treatments must account for nonmonospecific modalities. Future treatments must focus on combination modalities targeting the TME, such as coadministration of MSLN‐targeting drugs with therapies that deplete the TME or activate checkpoints to transform “cold” tumors into “hot” ones. In addition, advances in synthetic biology, such as tandem CARs, AdCAR‐Ts, IF‐THEN CARs, and nanocarriers for drug delivery, offer promising avenues for developing novel treatments that maximize antitumor activity while minimizing off‐target toxicity. The determination of the microenvironmental cues that drive the unique oncogenic activity of MSLN represents the basis of this engineering. In summary, the development of therapies targeting MSLN ultimately depends on optimizing patient selection using biomarker‐based stratification. Moving forward, there is a need to incorporate spatial transcriptomics, single‐cell RNA sequencing, and epigenetic profiling into clinical studies to precisely select patients whose tumors exhibit homogeneous MSLN expression and an immune‐friendly environment. By carefully dissecting MSLN context‐specific signaling pathways and engineering approaches to overcome impediments in the TME, MSLN‐targeting immunotherapies may become established treatment paradigms for drug‐resistant solid tumors.

## Author Contributions

Conceptualization and design: MKG, GPN, and RV. Writing – original draft: MKG. Writing – review and editing: MKG, GPN, and RV. All authors contributed to the article and approved the submitted version.

## Funding

The authors have nothing to report.

## Conflicts of Interest

The authors declare no conflicts of interest.

## Ethics Statement

This article involves only computational work and contains no studies with human participants or animals.

## Supporting information




**Supporting File 1**: mco271009‐sup‐0001‐Figure S1‐S11.pptx

## Data Availability

The authors have nothing to report.
